# The complex structure of bile salt hydrolase from *Lactobacillus salivarius* reveals the structural basis of substrate specificity

**DOI:** 10.1038/s41598-019-48850-6

**Published:** 2019-08-27

**Authors:** Fuzhou Xu, Xiao-Jian Hu, Warispreet Singh, Wenjing Geng, Irina G. Tikhonova, Jun Lin

**Affiliations:** 1 Department of Animal Science, The University of Tennessee, Knoxville, TN 37996 USA; 2 Institute of Animal Science and Veterinary Medicine, Beijing Academy of Agriculture and Forestry Sciences, Beijing, China; 3 State Key Laboratory of Genetic Engineering, School of Life Sciences, Collaborative Innovative Centre of Genetics and Development, Fudan University, Shanghai, 200438 China; 4 Molecular Therapeutics, School of Pharmacy, Medical Biology Centre, Queen’s University Belfast, BT9 7BL Northern Ireland, United Kingdom

**Keywords:** Bacterial techniques and applications, X-ray crystallography

## Abstract

The gut bacterial bile salt hydrolase (BSH) plays a critical role in host lipid metabolism and energy harvest. Therefore, BSH is a promising microbiome target to develop new therapies to regulate obesity in humans and novel non-antibiotic growth promoters for food animals. We previously reported the 1.90 Å *apo* crystal structure of BSH from *Lactobacillus salivarius* (*ls*BSH). In this study, we soaked the *ls*BSH crystal with glycocholic acid (GCA), a substrate, and obtained a 2.10 Å structure containing complex of *ls*BSH bound to GCA and cholic acid (CA), a product. The substrate/product sits in the water-exposed cavity molded by Loops 2 and 3. While the glycine moiety of GCA is exposed into a highly polar pocket, the sterane core of GCA is stabilized by aromatic and hydrophobic interactions. Comparison of product binding with BSH from *Clostridium perfringenes* reveals a distinct orientation of the sterane core in the binding site. The stability of the substrate-*ls*BSH complex and the putative catalytic mechanism were explored with molecular dynamics simulations. Site-directed mutagenesis of *ls*BSH demonstrated that Cys2 and Asn171 are critical for enzymatic activity, while Tyr24, Phe65 and Gln257 contribute to the substrate specificity. Together, this study provides structural insights into BSH-substrate interaction, the mechanism of catalysis and substrate specificity, which facilitate rational design of BSH inhibitors.

## Introduction

The bile salt hydrolase (BSH) produced by intestinal bacteria catalyzes deconjugation of glyco-conjugated and tauro-conjugated bile acids through the hydrolysis of the amide bond and the release of free bile acids (e.g. cholic acid and chenodeoxycholic acid) and amino acids (glycine and taurine)^1^. Deconjugation is a gateway reaction in the metabolism of bile acids in the small intestine^2,3^. Therefore, BSH participates in a range of metabolic processes in mammalians including the regulation of dietary lipid absorption, cholesterol metabolism, energy and inflammation homeostasis^4–6^. Modulation of BSH activity can affect host lipid metabolism, energy harvest and body weight gain, thus, is a promising strategy to control obesity in humans^7,8^ and to improve the growth performance in food animals^2^. Several BSH inhibitors showing a promise as animal growth promoters have been discovered in the high-throughput screening in our previous study^9^.

**Figure 1:**
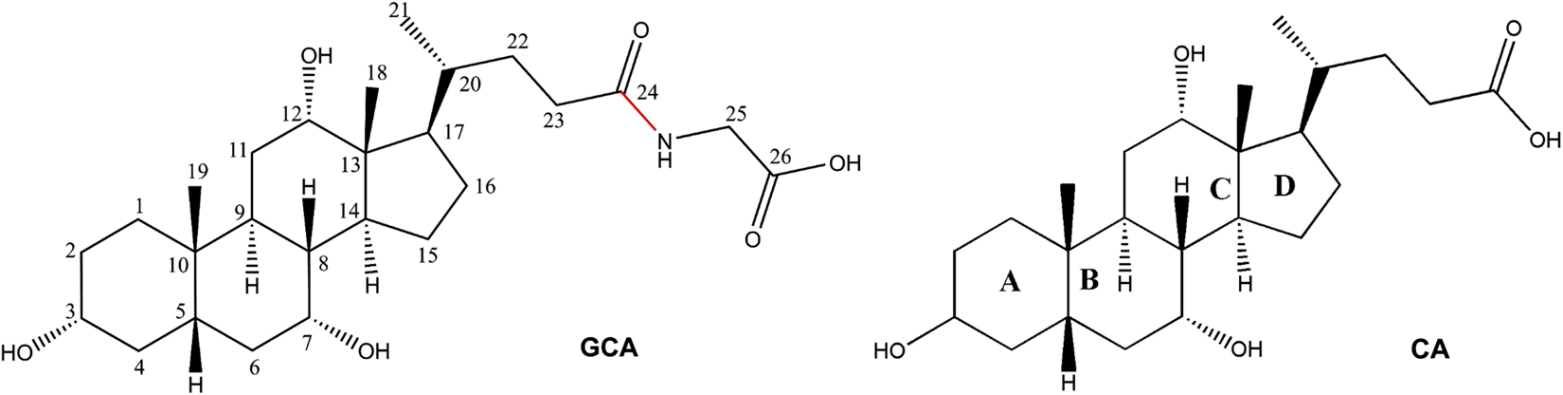
Chemical structure of GCA and CA. The carbons are numbered in GCA and the four rings (**A**–**D**) is marked in CA. The position of hydrolysis site (red line) is highlighted on the GCA molecule.

The BSH enzyme hydrolyses a substrate glycocholic acid (GCA) to product of cholic acid (CA) and glycine (Fig. 1). It is an N-terminal nucleophilic (Ntn) hydrolase^10^, which is known to undergo an autocatalytic cleavage of the N-terminal residue to expose the cysteine residue to act as a nucleophile^11^. Four crystal structures of BSH enzymes from *Enterococcus faecalis* (*ef*)^12^, *Bifidobacterium longum* (*bl*)^13^, *Clostridium perfringenes* (*cp*)^14^ and *Lactobacillus salivarius* (*ls*)^15^ are available in the literature. While the *ef*BSH, *bl*BSH and *ls*BSH are determined in the ligand-free form, the *cp*BSH complex with products, deoxycholate and taurine has provided the first structural basis of ligand interactions in BSH. Apart from the catalytic Cys2, the crystal structure of *cp*BSH indicates that conserved Arg18, Asp21, Asn82 and Arg228 are important in catalysis and substrate binding^14^. However, the molecular basis of substrate recognition and mechanism of inhibition is still elusive.

The sequence identity among the four crystallized BSH isotypes is only 30–54%. Indeed, our previous *ls*BSH crystal structure highlighted differences in the loops surrounding the active site^15^. It is apparent that binding of substrates has distinct structural features in BSH isotypes. To investigate the variations in substrate specificity, we soaked *ls*BSH with the GCA substrate to obtain a crystal structure of the protein-ligand complex. Thus, we here report the first structure of the *ls*BSH-GCA-substrate complex and the structure of the *ls*BSH-CA-product complex. The stability of the *ls*BSH-substrate complex and the putative catalytic mechanism were further explored in a molecular dynamics (MD) simulation study. We then validate the importance of several active site residues in substrate recognition and catalysis through mutagenesis and BSH activity analysis against six conjugated bile acids. Our work points into the molecular basis of substrate recognition and specificity in BSH isotypes.

## Results

### The overall structure of *ls*BSH bound to GCA or CA

**Figure 2:**
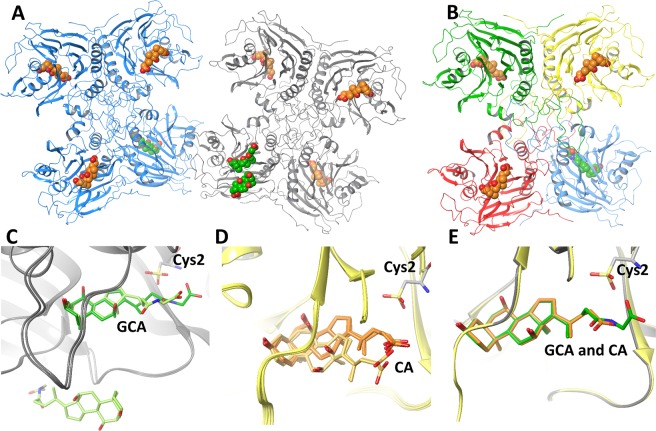
The overall structures of GCA-soaked *ls*BSH complex. (**A**) It consists of two tetramers, which are composed of chains A, B, C, D, E, F, G and H. In each *ls*BSH tetramer, one monomer is in complex with the substrate, GCA (in green), while the remaining monomers are in complex with the product, CA (in orange). (**B**) The tetramer structure formed by chains E, F, G and H. Each monomer has different color. (**C**) The superimposition of *ls*BSH monomers bound to GCA. GCA is in green from chain F and light green from chain A. The monomer of chain A contains the second GCA molecule at the vicinity of the active site. (**D**) The superimposition of *ls*BSH monomers bound to CA. CA is in orange from chains B, D, G and H and light orange from chains C and E. (**E**) The superimposition of *ls*BSH monomers bound to GCA and CA from chains F and B. The catalytic cysteine residue, Cys2 found in the oxidized form is shown in stick representation.

The 2.1 Å X-ray crystal structure of *ls*BSH bound to GCA and CA was obtained by soaking *apo ls*BSH crystals in the crystallization buffer containing 5 µM GCA for 4 hours. The obtained crystal structure revealed two tetramers (chains A-D formed one tetramer and chains E-H formed another one) in one asymmetric unit (Figs 2 and S1). The crystallographic parameters of the GCA-soaked *ls*BSH are shown in Table 1. Each *ls*BSH monomer contains two antiparallel β-sheets sandwiched between α-helices, adopting a four-layered αββα fold of the Ntn hydrolases. Further, the general tetrameric arrangement of monomers is consistent with the physiological assembly described for BSH^12^ and other Ntn hydrolase family members^16,17^. Apart from the N-terminus methionine residue, all amino acid residues of *ls*BSH were resolved in the crystal structure. The catalytic cysteine residue (Cys2) was found in the oxidized sulfonic form in all monomers (Fig. 2). Structural comparison of the eight monomers revealed identical protein conformations with the average root-mean-square deviation (RMSD) of 0.38 Å over 237 C_α_ atoms.

**Table 1:** Crystallographic parameters of the GCA-soaked *ls*BSH. Statistics for the highest-resolution shell are shown in parentheses.

Data Collection and Processing	
Space Group	P2_1_ (PDB ID 5Y7P)
Unit Cell	84.01, 94.09, 166.97, 90.0, 90.64, 90.0
Wavelength (Å)	1.5418
Resolution range (Å)	50.0–2.10 (2.14–2.10)
Completeness (%)	95.1 (89.7)
Mosaicity	0.947
I/σ (I)	12.7 (5.84)
Redundancy	5.3 (4.9)
*R*_meas_	0.078 (0.291)
Overall *B* factor from Wilson plot (Å^2^)	18.4
**Refinement statistics**	
Resolution range (Å)	28.39–2.101 (2.176–2.101)
Unique reflections	143212 (13183)
R_work_/R_free_	0.1796/0.2195
No. of non-H atoms	
Protein	20666
Ligand	309
Solvent	989
Total	21964
Solvent, %	45.5
R.m.s. deviations	
Bonds (Å)	0.008
Angles (°)	1.11
Average *B* factors (Å^2^)	
Overall	28.11
Protein	28.01
Ligands	36.76
Solvent	27.56
Ramachandran plot	
Most favoured (%)	96.64
Allowed (%)	3.20
Disallowed (%)	0.16

The *ls*BSH monomers were obtained bound to substrate GCA or product CA (Fig. 2C,D), revealing that GCA has been hydrolyzed by the enzyme to CA and glycine during soaking experiments. In each *ls*BSH tetramer, only one monomer is in complex with GCA (chains A and F), while the remaining monomers are in complex with CA. The RMSD of GCA atoms in the active site is 2.27 Å between two superimposed *ls*BSH monomers of chains A and F (Fig. 2C). The large deviation is due to a different position of the glycine and 3-methylpropyl carboxyl moieties of GCA. In chain F, the carbon of the amide bond is at the distance of 4.9 Å to the sulphur atom of the catalytic cysteine, while in chain A, this distance is 8.1 Å and the glycine moiety is in a more solvent-exposed conformation. Interestingly, the monomer belonging to chain A contains a second GCA molecule at the vicinity of the active site (Fig. 2A,C).

The four CA molecules in chains B, D, G and H occupy an identical position with the average RMSD of 0.43 Å, while the other two CA molecules of chains C and E are shifted towards a solvent exposed area with the average RMSD of 1.95 Å (Fig. 2D) to the cluster of four CA molecules. Comparison of GCA and CA positions in the *ls*BSH monomers shows that GCA of chain F and CA molecules of chains B, D, G and H have the smallest RMSD of 0.38 Å (Fig. 2E). The notable differences in positions of GCA and CA in several *ls*BSH monomers could be due to capturing different intermediate states of the ligands in the binding site or limitations of the soaking experiments.

### Interactions of *ls*BSH with GCA and CA in the crystal structures

**Figure 3:**
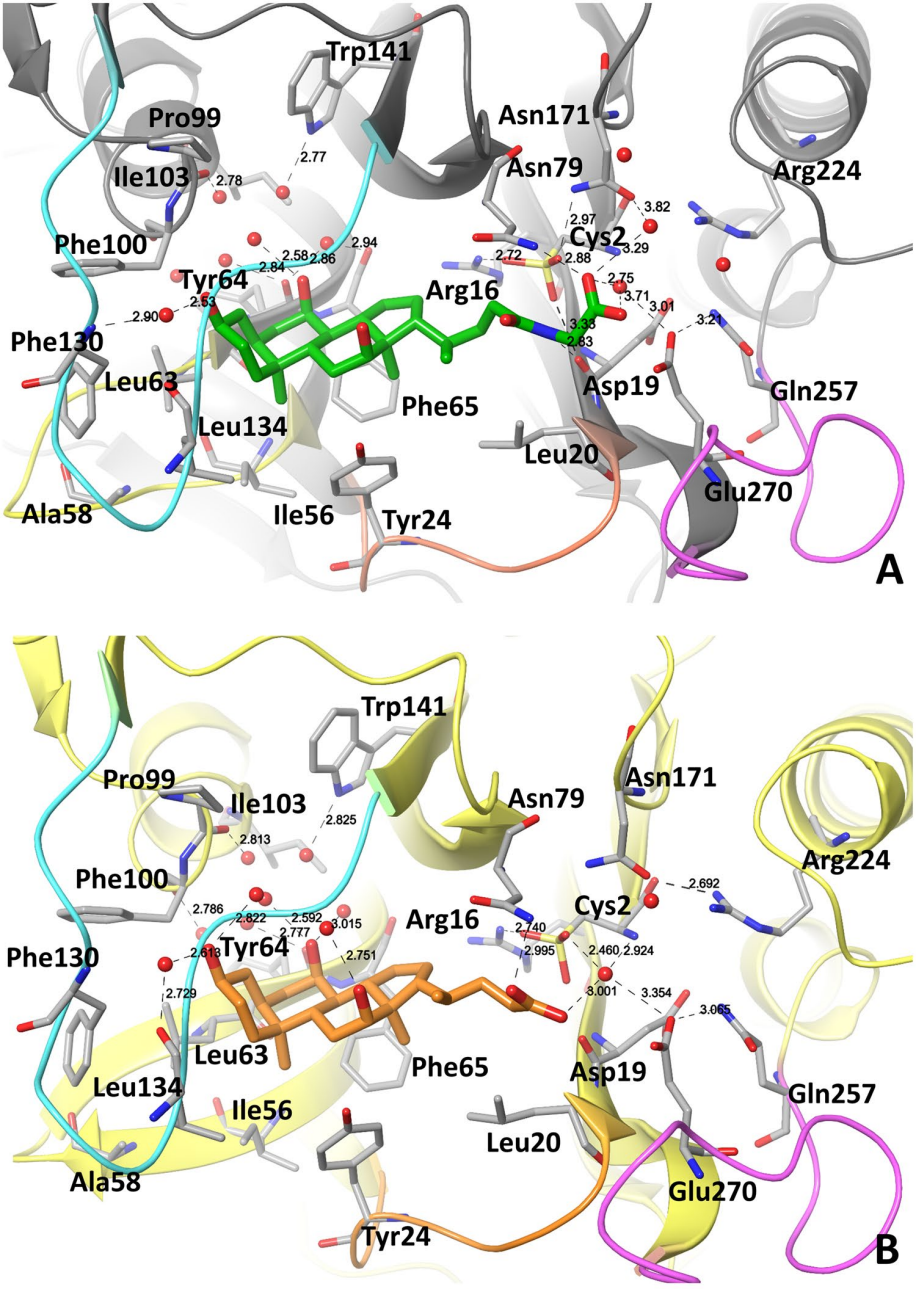
The *ls*BSH-GCA and *ls*BSH-CA complex structure. (**A**) Binding mode of GCA in the active site of *ls*BSH from Chain F. (**B**) Binding mode of CA in the active site of *ls*BSH from Chain B. The residues lining the active site are shown in stick representation. Oxygen atoms of water molecules within the active site are shown in red balls. GCA and CA are in green and orange, respectively. Loop 1 (residues: 21–26), Loop 2 (residues: 56–63), Loop 3 (residues:127–138) and Loop 4 (residues: 257–268), lining the active site are shown in orange, yellow, cyan and pink, respectively. Distances of polar contacts in Å are shown in dashed black lines.

The active site in *ls*BSH is located in a shallow and water-exposed cavity formed by β-sheets and four loops, Loops 1–4 (Figs 2C and 3). Loops 2 and 3 shape and partially close the active site of *ls*BSH from water exposure (Fig. 3). We chose the *ls*BSH complex bound to GCA from chain F to analyze the ligand-protein interactions since the glycine and 3-methylpropyl moieties of GCA occupy the catalytic site.

The glycine moiety of GCA points toward a highly polar area composed of Cys2, Arg16, Asp19, Asn79, Asn171, Arg224, Gln257 and Glu270 (Fig. 3A). The carboxyl group of the glycine moiety forms a H-bond with Asn79 and a water-mediated H-bond with Asn171. The amide bond of GCA is at the distance of 4.75 Å from the sulfur atom of the oxidized Cys2 (OCS-2). The NH-group of the GCA amide bond forms a H-bond with the backbone of Asp19 and the oxygen of OCS-2. In the case of four *ls*BSH monomers bound to CA (Chains B, D, G and H), the hydrolyzed carboxyl group is close to Asp19 and OCS-2 (Fig. 3B).

The oxidized catalytic Cys2 is engaged in a network of polar interactions (Figs 3A and S1). The terminal amino group of Cys2 forms polar interactions with Asp19 and Asn171 and a water-mediated H-bond with Glu270. The carbonyl group of Cys2 has a H-bond with Arg224. The OCS-2 is engaged in polar interactions with Arg16, the backbone of Asp19 and Asn79, the side chain of Asn171 and through a water-mediated interaction with Glu270. Comparative sequence analysis shows that Arg16, Asp19, Asn79, Asn171 and Arg224 are conserved in BSH isotypes (Fig. S2).

In the crystal structure, a water molecule that forms water-mediated H-bonds with Asp19, Glu270 and Cys2 (Figs 3A and S1) appears to facilitate the catalysis and hydration of the amide bond. The water molecule at the same position is also observed in the structure of *ls*BSH bound to CA, where it forms H-bonds with CA, OCS-2 and Asn171 (Fig. 3B).

The sterane core of GCA and CA is in the hydrophobic pocket composed of Leu20 and Tyr24 from Loop 1; Ile56 and Leu63 of Loop 2; Phe65 of the β-sheet; Leu134 and Phe130 of Loop 3; and Phe100 the α-helix top (Fig. 3). The α-surface of the sterane core faces Loop 3, whereas the β-surface of the sterane core that has two methyl groups faces Loops 1 and 2. The OH-group of sterane ring A forms a water-mediated H-bond with the backbone of Leu134, whereas the OH-group of sterane ring B forms a two-water-mediated H-bond with the side-chain of Trp141. This network of H-bonds is observed in both GCA- and CA- *ls*BSH complexes. The sterane core fits into the hydrophobic pocket, however, the pocket becomes hydrophilic the deeper it gets. Indeed, several water molecules are located at the bottom of the hydrophobic pocket in all *ls*BSH monomers, forming H-bonding with Tyr64, Pro99, Phe100, Ile103 and Trp141 (Fig. 3).

### Comparison of crystal structures of *ls*BSH and *cp*BSH bound to catalytic reaction products

**Figure 4:**
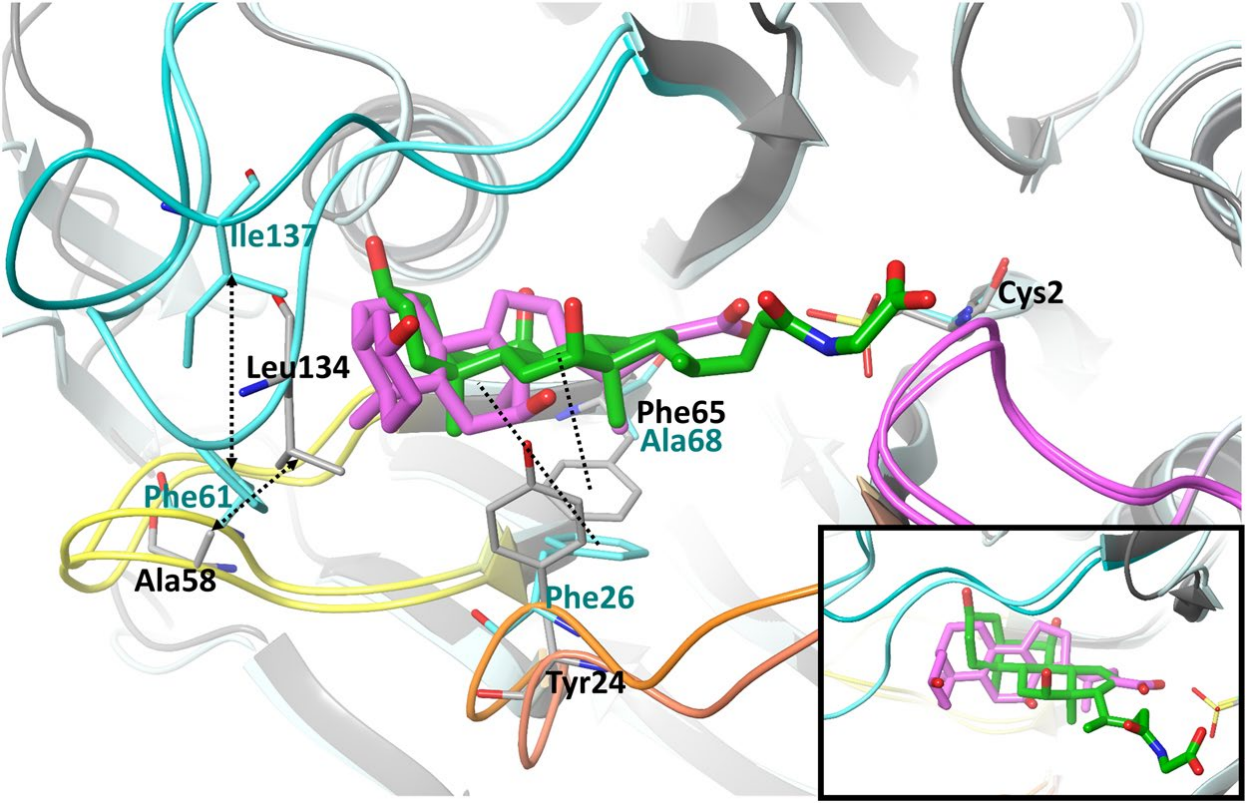
The superimposition of *ls*BSH and *cp*BSH active sites. The crystal structure of *cp*BSH in complex with deoxycholate, a product (PDB code: 2BJF) was used to superimpose with *ls*BSH bound to GCA, Chain F. Deoxycholate and GCA are in pink and green, respectively. The catalytic cysteine residue, Cys2 in the oxidized from is in stick representation. Loops 1–4 are shown in orange, yellow, cyan and pink, respectively. The non-conserved residues contributing to distinct substrate binding modes between *ls*BSH and *cp*BSH are shown in stick representation. Interactions between Ala58 of Loop 2 and Leu134 of Loop 3 in *ls*BSH and the corresponding pair of residues, Phe61 of Loop 2 and Ile137 of Loop 3 in *cp*BSH are shown in arrow dashed black lines. Hydrophobic interactions with Phe65 in *ls*BSH and Phe26 in *cp*BSH are shown in a dashed black line. A zoomed position of ligands is shown at the bottom right corner to highlight the distinct orientation of the sterane core in BSH isotypes. Amino acid residue labels in black and cyan are for *ls*BSH and *cp*BSH, respectively.

The crystal structure of *cp*BSH in the ligand-bound form, complexed with product deoxycholate, is available in the literature^14^. Superimposition of the *cp*BSH and *ls*BSH complexes shows that the orientation of the deoxycholate molecule in the *cp*BSH binding site is distinct from the positions of GCA or CA in the *ls*BSH binding site (Fig. 4). Thus, the α-face of the sterane core of deoxycholate is almost in the perpendicular orientation relative to the α-face of the sterane core of GCA and has a one-ring shift towards a water-exposed area in *cp*BSH. The different position of the sterane core is due to different conformations of Loops 2 and 3 that change the size of the binding groove in *cp*BSH and *ls*BSH. In *ls*BSH, hydrophobic interactions between Ala58 of Loop 2 and Leu134 of Loop3 pack the loops close to each other, pushing the sterane core of GCA and CA deeper to the binding groove (Fig. 4). In the case of *cp*BSH, the interactions between Loops 2 and 3 happen through bulky Phe61 (corresponding to Ala58) and Ile137 (corresponding to Leu134), which hold the loops in a more open conformation, leaving a room to occupy a water-exposed hydrophobic pocket.

Other differences in the binding site of BSH isotypes are linked to Loop 1. In *cp*BSH, Loop 1 provides hydrophobic force to interact with the lateral of sterane. In particular, the benzene ring of Phe26, a main hydrophobic contact of Loop 1, restrains the lateral of the sterane core in the *cp*BSH binding groove. At the corresponding site in *ls*BSH, the phenol ring of Tyr24 flips up on 90 degrees and the major hydrophobic interaction comes from Phe65 (corresponding to Ala68 in *cp*BSH) that holds the lateral of the sterane (Fig. 4) in the *ls*BSH binding groove.

### Refinement of the *ls*BSH-GCA complex with molecular dynamic simulations to reveal a catalytic geometry

BSHs undergo an auto-catalytic post-translational modification, which makes the Cys residue as the N-terminal residue^10^. The recent quantum mechanics and molecular mechanics (QM/MM) study of *cp*BSH in complex with tauro-deoxycholate has shown that Cys2 exists in the zwitterionic state, where the amino group is protonated and the thiol group is deprotonated^11^. This ionized state of Cys2 is stable by ~15 kcal/mol in comparison with the neutral form^11^. It appears that the conserved residues corresponding to Arg16, Asn79 and Asn171 in *ls*BSH are in the close proximity to Cys2 (2.7 Å) and reduces the pKa of the thiol group, making it easier to exist as a thiolate anion. Furthermore, the QM/MM study has also demonstrated that the thiolate anion of Cys2 performs a nucleophilic attack at the carbonyl carbon of the substrate amide bond. Taking advantage of the first *ls*BSH crystal structure in complex with the GCA substrate, we have further explored substrate interactions in the catalytic site in the presence of the Cys2 zwitterionic state using conventional atomistic MD simulations.

**Figure 5:**
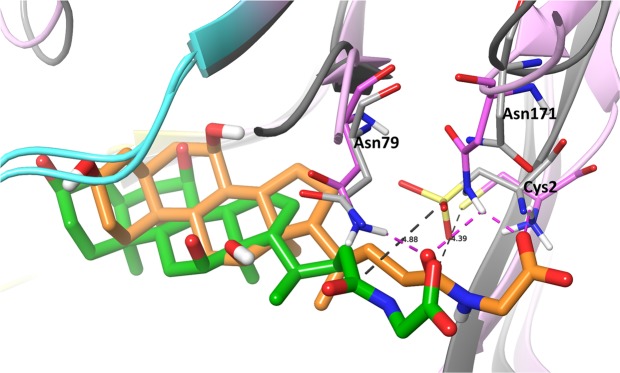
The superimposition of the *ls*BSH crystal structure and the molecular dynamics simulation representative structure. MD simulations of the crystal structure with Cys2 in the catalytically active zwitterionic state has been conducted to refine substrate interactions. In the simulations, the amide bond of GCA is pointed towards the oxoanion hole residues, Asn79 and Asn171 forming hydrogen bonds. GCA from the crystal structure and MD simulation conformations is in green and orange, respectively. The *ls*BSH crystal structure and MD conformation are in grey and pink, respectively. Cys2, Asn79 and Asn171 are in stick representation. Hydrogen bonds are in a dashed pink line. Distance between the carbon atom of the amide bond and the sulfur atom of Cys2 in Å are shown in a dashed black line.

The superimposition of the *ls*BSH crystal structure and the MD simulation representative structure is shown in Fig. 5. The thiolate anion comes closer to the carbonyl of the amide bond with the average distance of 4.4 Å. The notable change, we observe, in the simulations is the flip of the carbonyl group of the amide bond from the solvent-exposed area towards Asn79 and Asn171 and formation of H-bonds with these residues (Fig. 5). In this position, the amide bond of the substrate establishes a catalytically productive conformation, where the carbonyl group is stabilized by interactions with the oxoanion hole residues (Fig. S3). This amide conformation is in agreement with the proposed hydrolysis mechanism of the tauro-conjugated bile acid from the QM/MM study of *ls*BSH performed by Lodola *et al*.^11^.

The thiolate anion and the ammonium cation of Cys2 are stabilized by electrostatic interactions with Arg16 and Asp19 and hydrogen bonds with Asn171 (Fig. S4). Mutations of residues in the corresponded positions in the Ntn hydrolases dramatically reduced the enzyme activity^16^. Overall, the glycine moiety of GCA has shifted deeper to the hydrophilic pocket of Cys2, Asn79, Asn171, Arg224 and Gln257 during the MD simulations.

In the simulations, several water molecules are often present in the vicinity of the amide bond of the substrate and the amino group of Cys2 (Fig. S5). Like in the hydrolysis of the amide bond in other Ntn hydrolases^17^, it is expected that a water molecule initiates the deacylation step of the hydrolysis, where the amino group of Cys2 abstracts a proton from the water molecule and the resultant hydroxyl anion then attacks the carbon of the carbonyl bond. The X-ray structure and the MD simulations are in good agreement with the hydrophobic interactions made by the side chain of Leu18, Leu20, Tyr24, Ile56, Phe65, Leu134 and Leu139 with the GCA molecule (Fig. S6).

### Enzymatic activity of BSH mutants against six conjugated bile acids

To further explore the contribution of amino acid residues lining the active site of *ls*BSH to the catalysis and substrate binding we have performed site-directed mutagenesis of Cys2, Tyr24, Phe65, Asn171, Gln257 and Glu270 (Table 2). Cys2 and Asn171 were chosen to further confirm the Ntn hydrolase catalytic mechanism in *ls*BSH. Tyr24 and Phe65 were used to validate their role in the stabilization of the sterane ring, while Gln257 and Glu270 for the binding of the glycine moiety. Although Cys2 and Asn171 are fully conserved, the remaining residues vary in the BSH isotypes, thus likely contributing to substrate specificity (Fig. S2).

**Table 2:** Comparison of BSH enzyme activity of various BSH mutants for different bile salts.

BSH	Relative BSH activity^a^					
	GCA	GDCA	GCDCA	TCA	TDCA	TCDCA
WT	100	100	100	100	100	100
Cys2Ser	—	—	—	3.02	—	—
Tyr24Phe	79.17	71.43	118.59	100	100	100
Phe65Ala	—	—	3.70	64.15	52.73	101.89
Asn171Ala	7.08	3.57	4.07	10.75	4.73	16.41
Gln257Ala	25.17	16.49	13.87	98.44	96.21	93.13
Glu270Ala	159.86	79.38	103.65	100.78	104.55	102.29
Gln257Ala Glu270Ala	27.21	11.34	6.93	35.16	11.36	16.79
Δ164–171	—	—	—	—	—	—

^a^The BSH activity was measured as µmol of amino acids released from the substrate per minute per mg of BSH (μmol/min/mg). The activity of following six bile salt substrates were tested: glycocholic acid (GCA), glycodeoxycholic acid (GDCA), glycochenodeoxycholic acid (GCDCA), taurocholic acid (TCA), taurodeoxycholic acid (TDCA), taurochenodeoxycholic acid (TCDCA). Experiments were carried out in triplicate. The numbers indicate the relative activity (%) of the activity of specific BSH mutant when comparing with that of wild-type BSH enzyme. “−” represents no significant difference when comparing with negative control.

The constructed plasmids containing these mutations are shown in Table S1. The mutant BSH enzymes were purified from corresponding *E*. *coli* hosts and then analyzed by SDS-PAGE gel to demonstrate the high purity of the produced BSH mutants (Fig. S7). Circular dichroism analysis of the purified BSH enzymes indicated that all the mutant BSH enzymes including the Δ164–171 deletion mutant (Fig. S7) were still properly folded when compared to the wild-type *ls*BSH (data not shown). Subsequently, the wild-type *ls*BSH and its mutants were subjected to BSH activity assay for six conjugated bile acids.

As expected, the mutation of Cys2 to serine completely knocks out the BSH activity towards all tested bile acids. The Cys2Ser mutant only displayed marginal BSH activity for TCA as compared to wild-type *ls*BSH (Table 2). The effect of Cys2Ser mutation in *ls*BSH further confirms that Cys2 is vital for the catalytic activity of BSH.

Mutation of Asn171 to alanine caused a dramatic decrease in the BSH activity for all six bile salts (Table 2). In particular, the enzyme activity for GCA, GDCA, GCDCA, and TDCA was substantially reduced compared to the wild-type *ls*BSH. The drastic effect of the Asn171Ala mutation was also expected as Asn171 forms a network of hydrogen bonding with GCA and stabilizes the tetrahedral intermediate as a part of the oxyanion hole (Fig. S3). Deletion of the adjacent residues of Asn171 (Δ164–171) completely abolished the BSH activity toward all tested bile acids (Table 2), highlighting the critical role of the region in BSH catalysis.

Replacement of Tyr24 with phenylalanine did not cause significant changes in BSH activity towards three tested tauro-conjugated bile acids (Table 2). However, the Tyr24Phe mutation led to significantly reduced hydrolysis activity for the substrates of GCA and GDCA. Interestingly, this mutation resulted in a slight increase in BSH activity for GCDCA (Table 2). In the X-ray structure of *ls*BSH-GCA, the side chain of Tyr24 was 4.7 Å away from the hydroxyl group at position C12 of GCA. However, during the MD simulations, we observed the presence of water-mediated hydrogen bonds between the side chain of Tyr24 and the C12 hydroxyl group of the GCA molecule (Fig. S8A). The mutation of the Tyr24 to phenylalanine would have disrupted these hydrogen bonds and hence explains the decrease in the enzyme activity. On the contrary, GCDCA substrate that lacks the hydroxyl group at the C12 position would have been better stabilized by the presence of a hydrophobic Phe24 residue, hence improving its enzyme activity (Fig. S8B).

The mutation of Phe65 to Alanine abolished the BSH activity for GCA and GDCA but led to marginal activity for GCDCA (Table 2). This finding is consistent with the structural role of this residue in the stabilization of the sterane core. The enzyme activity of the Phe65Ala mutant for TCA and TDCA decreased by 40–50% compared to wild-type *ls*BSH, but for TCDCA was not affected (Table 2), suggesting a different contribution of Phe65 to the binding of tauro-conjugated bile salts.

The Gln257Ala mutation had a different effect on glycol-conjugated and tauro-conjugated bile salts. Thus, Gln257Ala mutation did not show a dramatic difference in tauro-conjugated bile salts (TCA, TDCA, TCDCA), but the activity for glycol-conjugated bile salts was decreased significantly (Table 2). Since Gln257 forms a hydrogen bond with Glu270 in the crystal structure, we created a double mutation of Gln257 and Glu270 to further validate the effect of Gln257 on substrate specificity. This double mutant caused a notable decrease of enzyme activity, ranging from 65% to 95%, for all six substrates relative to wild-type *ls*BSH. Notably, the Glu270Ala mutation alone did not cause significant difference in BSH activity towards all six substrates. These results suggested that Gln257 and Glu270 contribute to the hydrolysis of bile salts and especially, Gln257 may play a differential role in glycol-conjugated and tauro-conjugated bile salt hydrolysis.

## Discussion

We report here the first crystal structure of *ls*BSH in complex of a GCA substrate. It shows the binding environment of the substrate amino acid moiety and helps to map the catalytic mechanism of hydrolysis. In particular, the H-bond network involving the glycine moiety of the substrate and *ls*BSH residues: Cys2, Arg16, Asp19, Asn79, Asn171 and Arg224 highlights the importance of these residues for the catalysis. The MD simulation refinement of the crystal structure in the presence of the zwitterionic state of Cys2 further indicates that the carbonyl group of the GCA amide bond is engaged in hydrogen bonding with Asn79 and Asn171. Interactions with these residues are important to stabilize the carbonyl group of the amide bond during the hydrolysis (Fig. S3). Thus, the catalytic mechanism involves two steps: (1) the nucleophilic attack of the carbonyl carbon of the amide bond by the thiolate anion of Cys2 and the proton abstraction from the ionized amino group of Cys2 by the lone pair of the nitrogen of the amide bond; (2) and the deacylation of the enzyme with a help of a water molecule to form the neutral form of the N-terminal Cys2 and cholic acid (Fig. S3). Asn79 and Asn171 are proposed to form an ‘oxyanion hole’ that stabilizes tetrahedral intermediate complexes in acylation and deacylation reactions (Fig. S3). Mutagenesis of Cys2 and Asn171 in this work and mutagenesis of Asn79 from literature confirm the essential role of these residues in the hydrolysis^11^.

The structure of *ls*BSH bound to GCA also is of help to begin an understanding of structural reasons of different substrate preferences in BSH isotypes. Here, we compared the ligand complex structures of *ls*BSH, with a broad substrate specificity and *cp*BSH, with a narrow substrate specificity and identified two reasons, which could affect the substrate binding profile. The first reason is due to the different position of the sterane ring of bile acids in the binding site. In *ls*BSH, Loop 3 makes the sterane ring to bind deeper to the groove due to more compact interactions with Loop 2. The non-conserved residues, Ala58 (Phe61 in *cp*BSH) in Loop 2 and Leu134 (Ile137 in *cp*BSH) in Loop 3 contribute to an inward conformation of Loop 3 in *ls*BSH and, therefore to the distinct orientation of the α- and β-faces of the sterane core. In addition, we note that the sterane ring has a different pattern of hydrophobic interactions between *ls*BSH and *cp*BSH due to non-conserved residue Tyr24 (Phe26 in *cp*BSH) in Loop 1 and Phe65 (Ala68 in *cp*BSH) of the β-sheet. Mutation of Tyr24 and Phe65 in *ls*BSH demonstrates the contribution of these residues to substrate specificity.

Comparison of the *ls*BSH binding site with the *ef*BSH and *bs*BSH structures shows that the position of Loop 3 in *ef*BSH is similar to *ls*BSH, whereas Loop 3 in *bs*BSH has, even more, a solvent-exposed conformation than *cp*BSH. It appears that the binding site of the sterane core in *ls*BSH and *ef*BSH, both with a broad substrate spectrum^12^ has a similar shape and is distinct from the binding site of *cp*BSH and *bs*BSH with a narrow substrate spectrum^14,15^. It is expected that the orientation of the sterane core in the binding site varies in BSH isotypes contributing to a substrate binding profile.

The second reason is due to the binding pocket of a substrate amino acid moiety. In particular, Gln257 in *ls*BSH provides different substrate specificity upon mutation to alanine. This residue doesn’t form direct interactions with GCA but forms an H-bond network with several residues of the pocket. Thus, mutation of this residue could change the H-bond network and subsequently the shape and hydrophilic property of the pocket, which affects differently the binding of glycol-conjugated and tauro-conjugated substrates. While Gln257 is conserved in *ls*BSH and *ef*BSH, the residue at this position is methionine in *cp*BSH and *bl*BSH, this further suggests the potential importance of this residue in substrate specificity (Fig. S2).

Phylogenetic analysis of BSH isotypes from various bacteria and with available substrate binding profile divides BSH isotypes into four clusters^18^. Interestingly, in each cluster, there are BSH isotypes with broad and narrow substrate specificity. The sequence alignment of several BSH isotypes (Fig. S2) with distinct substrate specificity shows low sequence identity and potential contribution of residues at various positions to substrate specificity in BSH isotypes. In this study, we have identified residues at several positions that are important for the substrate binding profile in *ls*BSH, providing the first structural basis of BSH substrate specificity. However, more efforts should be carried out in future to explore substrate specificity across different BSH isotypes. The structural information of substrate binding and specificity will facilitate a rational approach in the design of BSH inhibitors.

## Materials and Methods

### Bacterial strain, plasmid, and compound

Major bacterial strains and plasmids used in this study and their sources are listed in Table S1. Briefly, the *E*. *coli* recombinant strain JL885 containing pBSH expression vector (Table S1), constructed in our previous study^19^, was used for purification of wild-type *ls*BSH in this study. The pBSH plasmid bearing original *bsh* gene from *L*. *salivarius* NRRL B-30514 was used as parent vector for site-directed mutagenesis. All compounds were purchased from Sigma-Aldrich (St. Louis, MO, USA), which include ampicillin, glycocholic acid (GCA), glycodeoxycholic acid (GDCA), glycochenodeoxycholic acid (GCDCA), taurocholic acid (TCA), taurodeoxycholic acid (TDCA), taurochenodeoxycholic acid (TCDCA).

### Macromolecule production and crystallization

The *ls*BSH protein was expressed, purified and crystallized as described in our recent report^15^. After purification and concentration, 16 mg/ml *ls*BSH in the buffer consisting of 10 mM sodium acetate (pH5.5), 400 mM NaCl, 1 mM DTT, 1 mM EDTA, 10% (v/v) glycerol was crystallized in the crystallization buffer that is comprised of 20% (w/v) polyethylene glycol 3,350, 0.2 M KH_2_PO_4_ (pH4.8) at 20 °C. To prepare soaking solution, GCA was dissolved in DMSO at the concentration of 100 mM and then the stock solution was diluted to final concentration of 10 mM using the crystallization buffer. Subsequently, to perform soaking experiment, one drop of 1.0 µl soaking solution was added beside the crystallization drop in which the *ls*BSH crystals had grown 3–5 days; a thin line was drawn between two drops to allow GCA diffuse slowly into crystals. Following 4 hr of the interaction of the two drops, the soaked crystals were harvested and flash frozen in crystallization buffer with additional 25% glycerol for diffraction experiment.

### Data collection and processing

The *apo ls*BSH-substrate complex crystal was flash frozen in the crystallization buffer with additional 25% glycerol as cryoprotectant and the diffraction data were collected at Biortus company (Jiangyin, China) with a home source diffraction system of Rigaku F-RE^++^/Saturn 944 CCD. Molecular replacement was performed with PHASER^20^ using our *apo*-*ls*BSH (PDB ID 5HKE) as an initial searching model. Structure refinement was performed with PHENIX^21^, REFMAC^22^, CCP4 Package^23^ and COOT^24^. The structures were drawn using Maestro 2018-4^25^ and PyMol^26^. The atomic coordinate and structure factor has been deposited in the Protein Data Bank (PDB ID 5Y7P).

### Site-directed amino acid substitution mutagenesis

The specific amino acid or region was mutated using QuickChange II site-directed mutagenesis kit (Agilent Technologies, USA). Briefly, the partial overlapping primer pairs containing the desired mutations (Table S2) were used for PCR amplification with the pBSH plasmid as template. The cycling conditions were as follows: 95 °C for 30 sec, followed by 12–18 cycles at 95 °C for 30 sec, 55 °C for 1 min, and 68 °C for 6.5 min, and finally at 4 °C. The PCR products were treated with *Dpn*I at 37 °C for 1 hr to digest the methylated, non-mutated parental DNA template. The digested products were transformed into XL1-Blue super competent cells and cell cultures were grown on LB agar plates with ampicillin (100 μg/ml). Plates were incubated at 37 °C overnight. In the following steps, single colony was incubated for plasmid DNA extraction. The recombinant plasmid with a specific amino acid substitution in pBSH was verified by sequencing (University of Tennessee Genomic Core). Total eight plasmids with different amino acids substitutions or deletion were generated and then transformed into *E*. *coli* BL21(DE3) competent cells to create the constructs producing BSH mutants (Table S1). These constructs and the control strain JL885 were used for purification of recombinant BSH enzymes as detailed in our recent publication^15^. Sodium dodecyl sulfate-polyacrylamide gel electrophoresis (SDS-PAGE) with a 12% (w/v) polyacrylamid separating gel was performed to monitor production and purification of the rBSH. The purified rBSH was finally dialyzed against PBS buffer containing 10% of glycerol and 5 mM of L-glutathione (pH 7.0). To determine if the BSH mutants are natively folded, circular dichroism experiment was performed using Aviv 202 CD spectrophotometer in Bioanalytical Resource Facility at The University of Tennessee (Knoxville, USA). The rBSH aliquots were stored in −80 °C freezer prior to use. Protein concentration was measured by BCA protein assay kit (Pierce).

### BSH activity assay

The wild-type *ls*BSH and corresponding mutants were subjected to the two-step standard BSH activity assays as described previously^11,16^ with brief modification. Briefly, 10 μl of purified BSH enzyme (1 µg/µl), 10 μl of conjugated bile salt (GCA, GDCA, GCDCA, TCA, TDCA, or TCDCA), 178 µl of reaction buffer (0.1 M sodium-phosphate, pH 6.0), and 2 μl of 1 M DTT were mixed gently and incubated at 37 °C for 30 min. Following the incubation, reaction tubes were put on ice to stop the reaction. Then, 50 µl aliquot of the reaction mixture was mixed with 50 µl 15% trichloroacetic acid (w/v) to fully stop the reaction, and the sample was centrifuged at 12,000 *g* for 5 min to remove the precipitate. The supernatant was mixed thoroughly with 950 μl ninhydrin reaction mix (250 μl of 1% ninhydrin [w/v], 100 μl of 0.5 M sodium-citrate buffer [pH5.5], and 600 μl of glycerol) and incubated in boiling water for 14 min. The reactions were stopped by putting reaction tubs on ice for 3 min and the absorbance of reaction mix at 570 nm wavelength was measured using Smart Spec Plus spectrophotometer (Bio-Rad). Standard curves using glycine or taurine were determined for each independent assay. All assays were performed in triplicate. Enzyme activity was expressed as 1 μmol of amino acids released from substrates per minute per mg of BSH^19^ and mutants relative activity compared to wild-type *ls*BSH was also calculated. Comparison of enzyme activity between mutants was tested by ANOVA. Levels of significance for *P* value were 5% (0.05). The statistical analysis was performed using SAS software (v9.03, SAS Institute Inc., Cary, NC). Relative activity (%) was calculated by dividing the mean activity of specific BSH mutant to the mean activity of wild-type BSH and then multiplied by 100.

### Molecular dynamics simulations

Chain F of the *ls*BSH-GCA complex (PDB code: 5Y7P) was used to perform MD simulations. The protonation states of the titrable residue in the crystal structure of the *ls*BSH in complex with GCA were assigned using the H++ server^27^ at pH 6.0. The side chain of Cys2 residue was set in the zwitterionic state based on the previous literature^11^. The parameters of GCA were developed using Antechamber of Amber Tools 16.

The productive MD simulations was run in the NPT ensemble at 310 K for 50 ns using the GPU version of the PMEMD engine^28^ integrated with the Amber 16 package^29^. The AMBER-FB15 force field^30^ was used in the simulations. The TIP3P^31^ water model and 10 Na + ions were used to solvate the *ls*BSH-GCA complex using an octahedral box. The entire system was first subjected to energy minimization using the steepest descent method followed by the conjugate gradient algorithm for total of 4000 steps. The system was then subjected to the controlled heating from 0 to 310 K using a Langevin thermostat with a collision frequency of 1 ps^−1^ using a NVT ensemble for 400 ps. The protein and the GCA molecule were restrained using a harmonic potential of 50 kcal mol^−1^ Å during the heating cycle. The density and the dimension of the entire system was equilibrated using the NPT ensemble for 1 ns. The Berendsen barostat was used to maintain the pressure at 1 bar during the equilibration phase. The production MD was run in the NPT ensemble for 50 ns. The SHAKE algorithm was used to constrain all the bonds with hydrogen atoms^32^. The periodic boundary conditions were used with a cutoff radius of 8 Å and electrostatic energy calculations were performed using the particle mesh Ewald (PME) method^33^. The individual frames were saved every 20 ps during the production run. CPPTRAJ^34^ and VMD^35^ were used to analyze the MD trajectory. The images were made using Maestro 2018-4^25^ and UCSF Chimera^36^.

## Supplementary information

Supplementary Information
