# Supplementary material for: The complex structure of bile salt hydrolase from Lactobacillus salivarius reveals the structural basis of substrate specificity
Source: Sci Rep. 2019 Aug 27;9:12438. doi: 10.1038/s41598-019-48850-6 (PMC6711994; doi:10.1038/s41598-019-48850-6)

SUPPLEMENTARY INFORMATION

The complex structure of bile salt hydrolase from *Lactobacillus salivarius* reveals the structural basis of substrate specificity

Fuzhou Xu[1,2], Xiao-Jian Hu[3]*, Warispreet Singh[4], Wenjing Geng[1], Irina G. Tikhonova[4]*, and Jun Lin[1]*

[1]Department of Animal Science, The University of Tennessee, Knoxville, TN 37996. USA; [2]Institute of Animal Science and Veterinary Medicine, Beijing Academy of Agriculture and Forestry Sciences, Beijing, China; [3] State Key Laboratory of Genetic Engineering, School of Life Sciences, Collaborative Innovative Centre of Genetics and Development,, Fudan University, Shanghai 200438, China, [4] Molecular Therapeutics, School of Pharmacy, Medical Biology Centre, Queen's University Belfast, BT9 7BL, Northern Ireland, United Kingdom

***Corresponding author:**

Dr. Xiao-Jian Hu, State Key Laboratory of Genetic Engineering, School of Life Sciences, Collaborative Innovative Center of Genetics and Development, Fudan University, Shanghai, China; Email: xjhu@fudan.edu.cn

Dr. Irina G. Tikhonova, Molecular Therapeutics, School of Pharmacy, Medical Biology Centre, Queen's University Belfast, BT9 7BL, Northern Ireland, United Kingdom, Email: i.tikhonova@qub.ac.uk

Dr. Jun Lin, Department of Animal Science, The University of Tennessee, 2506 River Drive. Knoxville, TN, USA; Email: jlin6@utk.edu

Figure S1. The active *ls*BSH-GCA and *ls*BSH-CA complex structure. The wall-eye stereo presentation of the polar interaction around the oxidative cysteine sulfonic acid OCS-2 in chain F (A), substrate GCA in chain F (B) and product CA in chain G (C). The refined density map around OCS-2, GCA, CA and ligand-bound water molecules were shown. The 2FoFc map contoured at 1.0 σ (blue) and FoFc map contoured at ± 3.0 σ (green and red) were shown. For clarity residues 128-139 are hided in (B) and (C). The polar contacts were plotted as red dot line and the length below 3.20 Å were shown.

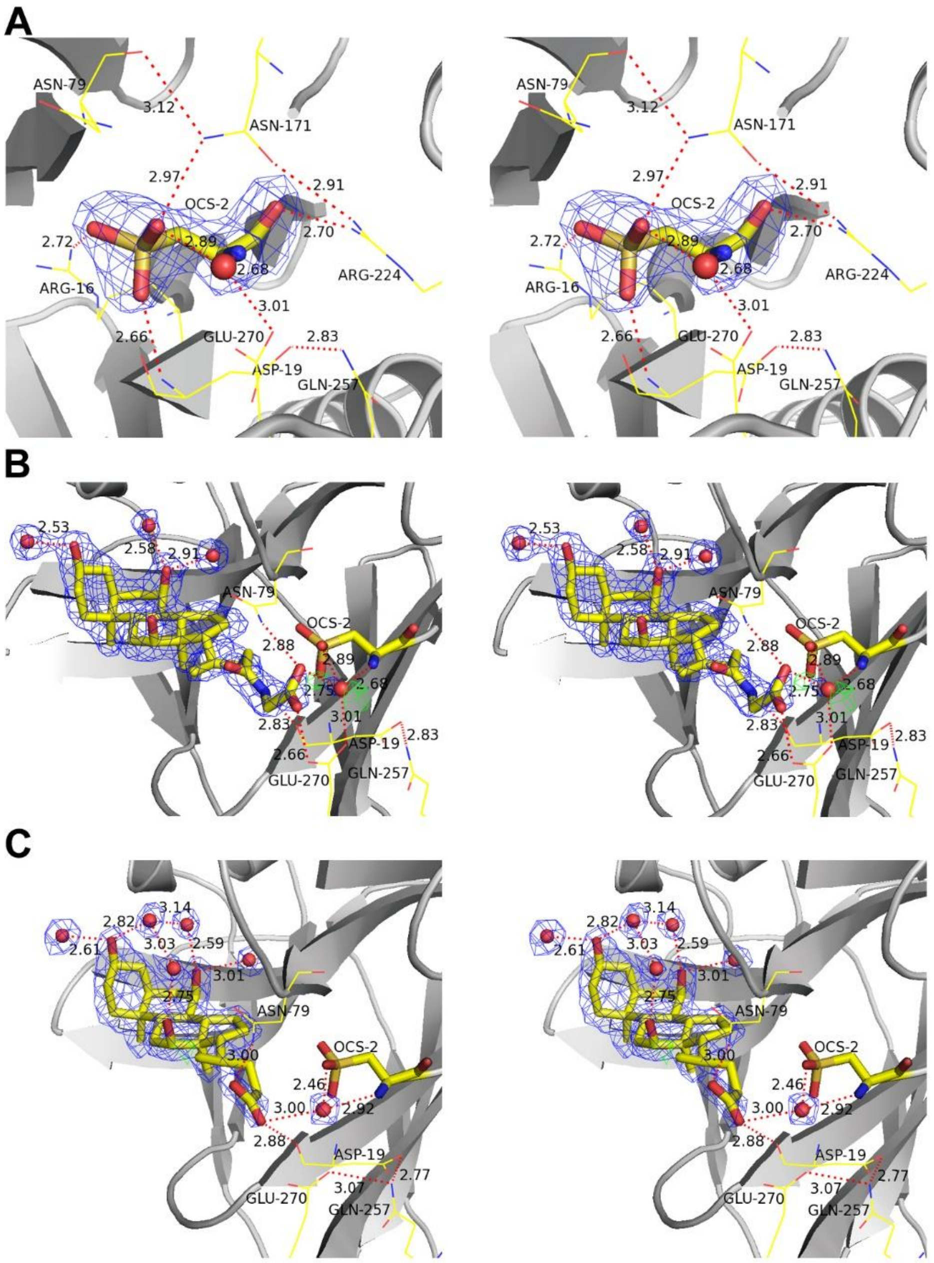

Figure S2. Sequence Alignment of BSH isotypes. The first four sequences of BSH with available crystal structures are shown with the PDB code. GenBank access code are given for all BSH sequences. Substrate preference: to glycol-conjugated bile acids, GC or tauro-conjugated bile acids, TC is shown from Dong *et al*, *Protein Science*, 2018, Vol. 27, pages 1742-1754. Loops 1-4 lining the active sites are highlighted in yellow. Residues involved in the catalytic reaction and substrate binding are in cyan and pink. Residues in pink contribute to substrate specificity. The red asterisk indicates the residues mutated in this study.

```
                                                              _loop_1_                                      _loop_2_                Substrate   GenBank code
                                  *       *        20        *   *         40         *         60         *  *
5Y7P_L_salivarius               : MCTAITLNGN--SNYFGRNLDLLDFSYGEEVIITPAEYEFKFRK-EKAIKNHKSLIGVGIVANDYPLYFDA :  67 GC      AFP87505.1
2BJF_C_Perfringens              : MCTGLALETKDGLHLFGRNMDIEYSFNQSIIFIPRNFKCVNKSNKKELTTKYAVLGMGTIFDDYPTFADG :  70 TC      AAC43454.1
4WL3_E_Feacalis                 : ~CTAITYVSK--DHYFGRNFDYEISYNEVVTITPRNYKFSFRE-VGNLDHHFAIIGIAAGIADYPLYYDA :  66 TC      EET97240.1
2HEZ_B__Longum                  : ~CTGVRFSDDEGNTYFGRNLDWSFSYGETILVTPRGYHYDTVFGAGGKAKPNAVIGVGVVMADRPMYFDC :  69 GC      AAF67801.1
BSHA_L_acidophilus_NCFM         : MCTSICYNPN--DHYFGRNLDYEIAYGQKVVIVPRNYEFKYRE-MPSQKMHYAFIGVSVVNDDYPLLCDA :  67 GC      AAV42751.1
BSHB_L_acidophilus_NCFM         : MCTSIIFSPK--DHYFGRNLDLEITFGQQVVITPRNYTFKFRK-MPSLKKHYAMIGISLDMDDYPLYFDA :  67 TC/GC   AAV42923.1
L_fermentum_NCDO394             : MCTSINVIAQDGYHVLGRTMDWDDLL-VSPIFTPRHYQLASVFDHRVHENPYAIIGGGSITERRTDVSDG :  69 GC      AEZ06356.1
BSHα_L_johnsonii100_100         : MCTSIVYSSN-NHHYFGRNLDLEISFGEHPVITPRNYEFQYRK-LPNKKAKYAMVGMAIVEDNYPLYFDA :  68 TC/GC   AAG22541.1
BSHA_L_johnsoni_PF01            : MCTSIVYSSN-NHHYFGRNLDLEISFGEHPVITPRNYEFQYRK-LPNKKAKYAMVGMAIVEDNYPLYFDA :  68 TC      EGP12224.1
BSHB_L_johnsoni_PF01            : MCTGLRFTDDQGNLYFGRNLDVGQDYGEGVIITPGNYPLPYKFLDNT-TTKKAVIGMGIVVDGYPSYFDC :  69 TC      EF536029.1
BSHC_L_johnsoni_PF01            : MCTSILYSPK--DNYFGRNLDYEIAYGQKVVITPRNYQLNYRH-LPTQDTHYAMIGVSVVANDYPLYCDA :  67 GC      EGP12391.1
L_plantarum_80                  : MCTAITYQSY--NNYFGRNFDYEISYNEMVTITPRKYPLVFRK-VENLDHHYAIIGITADVESYPLYYDA :  67 GC      AAB24746.1
L_plantaru_WCFS1                : MCTAITYQSY--NNYFGRNFDYEISYNEMVTITPRKYPLVFRK-VENLDHHYAIIGITADVESYPLYYDA :  67 GC      CAD65617.1
L_plantarum_80                  : MCTAITLNGN--SNYFGRNLDLLDFSYGEEVIITPAEYEFKFRK-EKAIKNHKSLIGVGIVANDYPLYFDA :  67 TC      ACL98197.1
L_plantarum_80                  : MCTSILYTAG--DHYFGRNLDLEISFGQQVVITPRDYPLNFRK-MPRLDHHYAITGMALVQDNYPLYFDG :  67 TC/GC   ACL98205.1
L_salivarius_JCM1046            : MCTAITLNGN--SNYFGRNLDLLDFSYGEQVIITPAEYEFKFRK-EKAIKNHKSLIGVGIVANAYPLYFDA :  67 TC      ACL98194.1
L_salivarius_UCC118             : MCTAITLNGN--NNYFGRNLDLLDFSYGEQVIITPAEYEFKFRK-EKAIKNHKSLIGVGIVANDYPLYFDA :  67 GC      ACL98201.1
L_salivarius_B-30514            : MCTAITYQSY--NNYFGRNFDYEISYNEMVTITPRKYPLVFRK-VENLDHHYAIIGITADVESYPLYYDA :  67 GC      AFP87505.1
BSHβ_L_johnsonii100_100         : MCTGLRFTDDQGNLYFGRNLDVGQDYGEGVIITPRNYPLPYKFLDNT-TTKKAVIGMGIVVDGYPSYFDC :  69 TC/GC   AAC34381.1
B_animalis_subsp.lactis_Bi30    : MCTAVRFDDGQNNMYFGRNLDWSEDYGEKIVFAPHDYHYAPAFNAEDKNH--PVIGIGIIVEDTPLYFDC :  68 GC      AEK27050.1
B_animalis_subsp.Lactis_KL612   : MCTAVRFDDGQNNMYFGRNLDWSEDYGEKIVFAPHDYHYAPAFNAEDKNH--PVIGIGIIVEDTPLYFDC :  68 GC      AAS98803.1
B_bifidum_ATTCC_11863           : MCTGVRFSDDEGNMYFGRNLDWSFSYGETILVTPRGYQYDYEYGAEGKSEPNAVIGVGVVMTDRPMYFDC :  70 GC      AAR39435.1
                                   2             16 19   24                                 58     65
```

```
                                                                      __loop_3______
                                     80         *       100         *       120         *       140
5Y7P_L_salivarius             : INEDGLGMAGLNFPGNAYYSDALENDKDNITPFEFIPWILGQCSDVNEARNLVEKINLINLSFS-EQLPL : 136
2BJF_C_Perfringens            : MNEKGLGCAGLNFPVYVSYSKEDIEGKTNIPVYNFLLWVLANFSSVEEVKEALKNANIVDIPIS-ENIPN : 139
4WL3_E_Feacalis               : INEKGLGMAGLNFSGYADYKKI-EEGKENVSPFEFIPWVLGQCSTVDEAKKLLKNLNLVNINFS-DELPL : 134
2HEZ_B__Longum                : ANEHGLAIAGLNFPGYASFVHEPVEGTENVATFEFPLWVARNFDSVDEVEETLRNVTLVSQIVP--GQQE : 137
BSHA_L_acidophilus_NCFM       : INEKGLGIAGLNFQGPNHYFPK-IEGKKNIASFELMPYLLSNCENTDDVKEILDNANILNISFS-ANYPA : 135
BSHB_L_acidophilus_NCFM       : TNEKGLGMAGLNYPGNATYYEE-KENKDNIASFEFIPWILGQCSTISEVKDLLSRINIADLNFS-EKMQA : 135
L_fermentum_NCDO394           : VNEFGLMAQKLTFKNGARLVDERHPDKVQLAAFELIFYLLGHFKSVADVAAHLDQIELMNDVNADVPFGY : 139
BSHα_L_johnsonii100_100       : SNEEGLGIAGLNFDGPCHYFPE-NAEKNNVTPFELIPYLLSQCTTVAEVKDALKDVSLVNINFS-EKLPL : 136
BSHA_L_johnsoni_PF01          : SNEEGLGIAGLNFDGPCHYFPE-VSGKNNVTPFELIPYLLSQYTTVAEVKEALKSVNLVKINFS-EKLQL : 136
BSHB_L_johnsoni_PF01          : YNEDGLGIAGLNFPHFAKFSDGPIDGKINLASYEIMLWVTQNFTHVSEVKEALKNVNLVNEAIN-TSFAV : 138
BSHC_L_johnsoni_PF01          : INEKGLGIAGLNFTGPGKYFAV-DESKKNVTSFELIPYLLSSCETIEDVKKLLSETNITDESFS-KDLPV : 135
L_plantarum_80                : MNEKGLCIAGLNFAGYADYKKY-DADKVNITPFELIPWLLGQFSSVREVKKNIQKLNLVNINFS-EQLPL : 135
L_plantaru_WCFS1              : MNEKGLCIAGLNFAGYADYKKY-DADKVNITPFELIPWLLGQFSSVREVKKNIQKLNLVNINFS-EQLPL : 135
L_plantarum_80                : INEDGLGMAGLNFPGNAYYSDALENDKDNITPFEFIPWILGQCSDVNEARNLVERINLINLSFS-EQLPL : 136
L_plantarum_80                : ANEEGLGMAGLNFDGPAHFFPV-EEGKDNVSPFEFIPYILGQCKNVAEAKELLKSLNLVNINFS-DQLQL : 135
L_salivarius_JCM1046          : INEDGLGMAGLNFPGNAYYSDALENDKDNITPFEFIPWILRQCSDVNEARNLVERINLINLSFS-EQLPL : 136
L_salivarius_UCC118           : INEDGLGMAGLNFPGNAYYSDALENDKDNITPFEFIPWILGQCSDVNEARNLVEKINLINLSFS-EQLPL : 136
L_salivarius_B-30514          : MNEKGLCIAGLNFAGYADYKKY-DADKVNITPFELIPWLLGQFSSVREVKKNIQKLNLVNINFS-EQLPL : 135
BSHβ_L_johnsonii100_100       : YNEDGLGIAGLNFPHFAKFSDGPIDGKINLASYEIMLWVTQNFTHVSEVKEALKNVNLVNEAIN-TSFAV : 138
B_animalis_subsp.lactis_Bi30  : MNDAGLAVAGLNFAKYCKYATEAVNFTTNVAAYEFPLWVTRNFTSVDDVQEALKNVTIVGKPIN-DRFPV : 137
B_animalis_subsp.Lactis_KL612 : MNDAGLAVAGLNFAKYCKYATEAVNFTTNVAAYEFPLWVTRNFTSVDDVQEALKNVTIVGKPIN-DRFPV : 137
B_bifidum_ATTCC_11863         : ANEHGLAIAGLNFPGYASFAHEPVEGTENVATFEFPLWVARNFDSVDEVEEALKNVTLVSQVVP--GQQE : 138
                                           79                                                134

                                __
                                           *       160       ********180         *       200         *
5Y7P_L_salivarius             : AGLHWLIADRE-KSIVVEVT-KSGVHIYDNPIGILTNNPEFNYQMYNLNKYRNLSISTPQNTFSDSVDLK : 204
2BJF_C_Perfringens            : TTLHWMISDITGKSIVVEQT-KEKLNVFDNNIGVLTNSPTFDWHVANLNQYVGLRYNQVPEFKLGDQSLT : 208
4WL3_E_Feacalis               : SPLHWLLADKE-QSIVVEST-KEGLRVFDNPVGVLTNNPTFDYQLFNLNNYRVLSTRTPKNNFSDQIELD : 202
2HEZ_B__Longum                : SLLHWFIGDGK-RSIVVEQM-ADGMHVHHDDVDVLTNQPTFDFHMENLRNYMCVSNEMAEPTSWGKASLT : 205
BSHA_L_acidophilus_NCFM       : ADLHWILSDKAGKSIVVEST-NSGLHIYDNPVNVLTNNPEFPDQLIKLSDYADVTPHNPKNTLVPNVDLN : 204
BSHB_L_acidophilus_NCFM       : SSLHWLIADKTGTSLVVETD-KDGMHIYDNPVGCLTNNPQFPKQLFNLNNYADVSPKMPKNNFSDKVNMA : 204
L_fermentum_NCDO394           : SEQHFVLSDPTGRCVVIEPS-EHPLKLIDNPLGIMTNMPKFDHQLERLQDYLDFTPDFLNGTLAPNTFHV : 208
BSHα_L_johnsonii100_100       : SPLHWLMADKTGESIVVEST-LSGLHVYDNPVHVLTNNPEFPGQLRNLANYSNIAPAQPKNTLVPGVDLN : 205
BSHA_L_johnsoni_PF01          : SPLHWLMADKTGESIVVEST-LSGLHVYDNPVHVLTNNPEFPGQLSNLANYSNIAPSQPKNTLVPGVDLN : 205
BSHB_L_johnsoni_PF01          : APLHWIISDSD-EAIIVEVSKQYGMKVFDDKVGVLTNSPDFNWHLTNLGNYTGLNPHDATAQSWNGQKVA : 207
BSHC_L_johnsoni_PF01          : TTLHWLMGDKSGKSIVIEST-ETGLHVYDNPVNTLTNNPVFPAQVETLANFASVSPAQPKNTLVPNADIN : 204
L_plantarum_80                : SPLHWLVADKQ-ESIVIESV-KEGLKIYDNPVGVLTNNPNFDYQLFNLNNYRALSNSTPQNSFSEKVDLD : 203
L_plantaru_WCFS1              : SPLHWLVADKQ-ESIVIESV-KEGLKIYDNPVGVLTNNPNFDYQLFNLNNYRALSNSTPQNSFSEKVDLD : 203
L_plantarum_80                : AGLHWLIADRE-KSIVVEVT-KSGVHIYDNPIGVLTNNPEFNYQMYNLNKYRNLSISTPQNTFSDSVDLK : 204
L_plantarum_80                : SPLHWLIADKSGAAITVEST-ASGLHVYDNPVNVLTNNPEFPDQLTNLANYQSVSPANPANTLAPQTALA : 204
L_salivarius_JCM1046          : AGLHWLIADRE-KSIVVEVT-KSGVHIYDNPIGVLTNNPEFNYQMYNLNKYRNLSISTPQNTFSDSVDLK : 204
L_salivarius_UCC118           : AGLHWLIADRE-KSIVVEVT-KSGVHIYDN--------PEFNYQMYNLNKYRNLSINTPQNTFSDSVDLK : 196
L_salivarius_B-30514          : SPLHWLVADKQ-ESIVIESV-KEGLKIYDNPVGVLTNNPNFDYQLFNLNNYRALSNSTPQNSFSEKVDLD : 203
BSHβ_L_johnsonii100_100       : APLHWIISDSD-EAIIVEVSKQYGMKVFDDKVGVLTNSPDFNWHLTNLGNYTGLNPHDATAQSWNGQKVA : 207
B_animalis_subsp.lactis_Bi30  : ATLHWIIADNT-RSIVVECT-EDGMHVYDDDVDVLTNQPPFPQQIEHLDNYAYVSPRTGKSVKWGSSELE : 205
B_animalis_subsp.Lactis_KL612 : ATLHWIIADNT-RSIVVECT-EDGMHVYDDDVDVLTNQPPFPQQIEHLDNYAYVSPRTGKSVKWGSSELE : 205
B_bifidum_ATTCC_11863         : SLLHWFIGDGT-RSIVVEQM-ADGMHVHHDDVDVLTNQPTFDFHMENLRNYMCVSNEMAEPTTWGKAELS : 206
                                                                     171
```

```
                                                                                      ___loop_4___
                                   220         *        240         *        260 *         *     * 280
5Y7P_L_salivarius                : VDGTGFGGIGLPGDVSPESRFVRATFSKLNSSKGMTVEEDITQFFHILGTVEQIKGVNKTESGKEEYTVY : 274
2BJF_C_Perfringens               : ALGQGTGLVGLPGDFTPASRFIRVAFLRDAMIKNDKDSIDLIEFFHILNNVAMVRGSTRTVEEKSDLTQY : 278
4WL3_E_Feacalis                  : IYSRGMGGIGLPGDLSSVSRFVKATFTKLNSVSRSSEYESISQFFHILSSVEQQKGLCDVGDEKYEYTIY : 272
2HEZ_B__Longum                   : AWGAGVGMHGIPGDVSSPSRFVRVAYTNAHYPQQNDEAANVSRLFHTLGSVQMVDGMAKMGDGQFERTLF : 275
BSHA_L_acidophilus_NCFM          : LYSRGLGTHHLPGGMDSSSRFVKVAFVLAHTPQGKNEVENVTNYFHILHSVEQPDGLDEVEDNRYEYTMY : 274
BSHB_L_acidophilus_NCFM          : GYSRGLGSHNLPGGMDSESRFVRVAFNKFNAPIAETEEENIDTYFHILHSVEQQKGLDEVGPNSFEYTIY : 274
L_fermentum_NCDO394              : TTGKLSGKKTPPGAYTPKGRYVRAAYMKELADQPASKDEALATTWHLLDSVTVPKSKA----HRPTFSVY : 274
BSHα_L_johnsonii100_100          : LYSRGLGTHFLPGGMDSASRFVKIAFVRAHSPQGNNELSSVTNYFHILHSVEQPKGTDEVGPNSYEYTIY : 275
BSHA_L_johnsoni_PF01             : LYSRGLGTHFLPGGMDSASRFVKVAFVRAHSPQGNNELSSVTNYFHILHSVEQPKGTDEVGPNSYEYTIY : 275
BSHB_L_johnsoni_PF01             : PWGVGTGSLGLPGDSIPADRFVKAAYLNVNYPTAKGEKANVAKFFNILKSVAMIKGSVVNDQGKDEYTVY : 277
BSHC_L_johnsoni_PF01             : LYSRGLGTHHLPGGTDSNSRFIKASFVLAHSPKGNDEVENVTNFFHILHSVEQAKGTDEVEDNVFEFTMY : 274
L_plantarum_80                   : SYSRGMGGLGLPGDLSSMSRFVRAAFTKLNSLPMQTESGSVSQFFHILGSVEQQKGLCEVTDGKYEYTIY : 273
L_plantaru_WCFS1                 : SYSRGMGGLGLPGDLSSMSRFVRAAFTKLNSLSMQTESGSVSQFFHILGSVEQQKGLCEVTDGKYEYTIY : 273
L_plantarum_80                   : VDGTGFGGIGLPGDVSPESRFVRAAFSKLNSSKGTTVEEDITQFFHILGTIEQIKGVNKTESGKEEYTVY : 274
L_plantarum_80                   : SYSRGAGSHFLPGGMDSESRFVKEVFTLQHAPAGETEVANVTNYFHCLHAVEQQKGLDEVGKDQFEYTIY : 274
L_salivarius_JCM1046             : VDGTGFGGIGLPGDVSPESRFVRAAFSKLNSSKGTTVEEDITQFFHILGTVEQIKGVNKTESGKEEYTVY : 274
L_salivarius_UCC118              : VDGTGFGGIGLPGDVSPESRFVRAAFSKLNSSKGTTVEEDITQFFHILGTVGQIKGVNKTESGKEEYTVY : 266
L_salivarius_B-30514             : SYSRGMGGLGLPGDLSSMSRFVRAAFTKLNSLSMQTESGSVSQFFHILGSVEQQKGLCEVTDGKYEYTIY : 273
BSHβ_L_johnsonii100_100          : PWGVGTGSLGLPGDSIPADRFVKAAYLNANYPTVKGEKANVAKFFNILKSVAMIKGSVVNDQGSDEYTVY : 277
B_animalis_subsp.lactis_Bi30     : TNQDSNSSQGLPGGYGSMARFVRAAYNNTHYPTQSGENANVNRLFKTLSTAAVIEGTAISANGEFEKTLF : 275
B_animalis_subsp.Lactis_KL612    : TNQDSNSSQGLPGGYGSMARFVRAAYNNTHYPTQSGENANVNRLFKTLSTAAVIEGTAISANAEFEKTLF : 275
B_bifidum_ATTCC_11863            : AWGAGVSMHGIPGDVSSPSRFVRVAYTNTHYPQQNNEAANVSRLFHTLVSVQMVDGMSKMGNGQFERTLF : 276
                                                     224                                 257         270

                                             *       300         *       320         *       340
5Y7P_L_salivarius                : SNCYDLDNKTLYYTTYENRQIVAVTLNK-DKDGNRLVTYPFERKQIINKLNLERHHHHHH : 333
2BJF_C_Perfringens               : TSCMCLEKGIYYYNTYENNQINAIDMNKENLDGNEIKTYKYNKTLSINHVN~~~~~~~~~ : 329
4WL3_E_Feacalis                  : SSCCNLEKGIYYYRTYDNSQITAVDMNKENLEKDSLIVYPMVETQQINYANLEHHHHHH~ : 331
2HEZ_B__Longum                   : TSGYSSKTNTYYMNTYDDPAIRSYAMADYDMDSSELISVAR~~~~~~~~~~~~~~~~~~~ : 316
BSHA_L_acidophilus_NCFM          : TDCMNLDKGILYFTTYDNNRINAVDMHKADLDSEDLICYDLFKKQDIEYMN~~~~~~~~~ : 325
BSHB_L_acidophilus_NCFM          : SDGTNLDKGIFYYTTYSNKQINVVDMNKEDLDSSNLITYDMLDKTKFNHQN~~~~~~~~~ : 325
L_fermentum_NCDO394              : RAATVAEDRTYYFQSYHQAQVTSVKLTDDLLKRATPLVFDTADVWAPVKLN~~~~~~~~~ : 325
BSHα_L_johnsonii100_100          : SDGTNLETGTFYYTNYENNQINAIELNKENLNGDELTDYKLIEKQTINYQN~~~~~~~~~ : 326
BSHA_L_johnsoni_PF01             : SDGTNLETGTFYYTNYENNQINAIELNKENLNGDELIDYKLIEKQTINYQN~~~~~~~~~ : 326
BSHB_L_johnsoni_PF01             : TACYSSGSKTYYCNFEDDFELKTYKLDDHTMNSTSLVTY~~~~~~~~~~~~~~~~~~~~~ : 316
BSHC_L_johnsoni_PF01             : SDCMNLDKGILYFTTYDNNQINAVDMNNENLDTSDLITYELFKDQAIKFEN~~~~~~~~~ : 325
L_plantarum_80                   : SSCCDMDKGVYYYRTYDNSQINSVNLNHEHLDTTELISYPLRSEAQYYAVN~~~~~~~~~ : 324
L_plantaru_WCFS1                 : SSCCDMDKGVYYYRTYDNSQINSVSLNHEHLDTTELISYPLRSEAQYYAVN~~~~~~~~~ : 324
L_plantarum_80                   : SNCYDLDNKTLYYTTYENRQIVSVTLNK-DKNGNKLVVYPFERKQIINKLN~~~~~~~~~ : 324
L_plantarum_80                   : SDGVNLTTGTFYYTTYDNNQINAVKMHAEDMEGQQLHRFPIASHQSINMQN~~~~~~~~~ : 325
L_salivarius_JCM1046             : SNCYDLDNKTLYYTTYENRQIVAVTLNE-DKNGNGLIAYPFERKQVINKLN~~~~~~~~~ : 324
L_salivarius_UCC118              : SNCYDLDNKTLYYTTYENRQIVAVTLNK-DKDGNRLVTYPFERKQIINKLN~~~~~~~~~ : 316
L_salivarius_B-30514             : SSCCDMDKGVYYYRTYDNSQINSVSLNHEHLDTTELISYPLRSEAQYYAVN~~~~~~~~~ : 324
BSHβ_L_johnsonii100_100          : TACYSSGSKTYYCNFEDDFELKTYKLDDHTMNSTSLVTY~~~~~~~~~~~~~~~~~~~~~ : 316
B_animalis_subsp.lactis_Bi30     : SDCYSTATQTVYLKKYDDMAVHSYAVKDFDASSNQLQSK~~~~~~~~~~~~~~~~~~~~~ : 314
B_animalis_subsp.Lactis_KL612    : SDCYSTATQTVYLKKYDDMAVHSYAVKDFDASSNQLQSK~~~~~~~~~~~~~~~~~~~~~ : 314
B_bifidum_ATTCC_11863            : TSGYSGKTNTYYMNTYEDPAIRSFAMSDFDMDSSELITAD~~~~~~~~~~~~~~~~~~~~ : 316
```

Figure S3. Proposed hydrolysis mechanism of GCA in BSH. (a) The neutral form of the N-terminal Cys2; (b) the zwitterionic state of the N-terminal Cys2; in this state the nucleophilic thiolate anion attacks the carbonyl carbon of the amide bond of GCA; (c) the proposed transition state associated with the nucleophilic attack by the thiolate anion and the proton abstraction by the lone pair of the nitrogen of the amide bond; (d) the acylated enzyme substrate complex and the free glycine amino acid; (e) proton abstraction from the water molecule by the N-terminal primary amine; (f) nucleophilic attack of the hydroxyl group to the carbonyl carbon of the acylated enzyme substrate complex, (g) the proposed transition state associated with the nucleophilic attack by the hydroxyl anion and proton abstraction by the sulphur atom of Cys2, (i) the neutral form of the N-terminal Cys2 and cholic acid. The lone pair of the electrons are represented by the double dots; breaking and forming bonds are in dashed lines. The protein residues, the glycine fragment of GCA and the water molecule are shown in black, pink and blue, respectively.

a

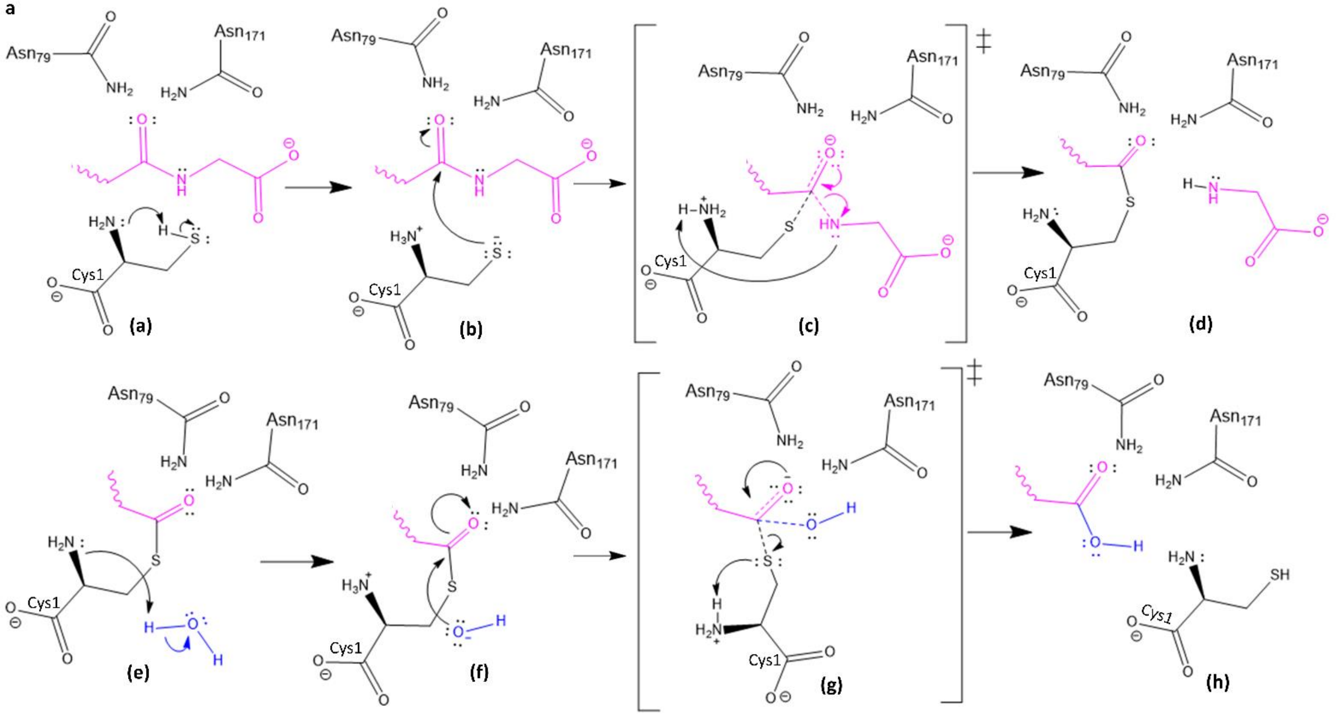

Figure S4. The active site of the *ls*BSH in complex with GCA. The snapshot of the most populated cluster obtained from the 50ns MD simulation. The GCA molecule is colored yellow and the active site residues are shown in grey stick representation. The loop region consisting of residues 164-171 are shown in red ribbon representation.

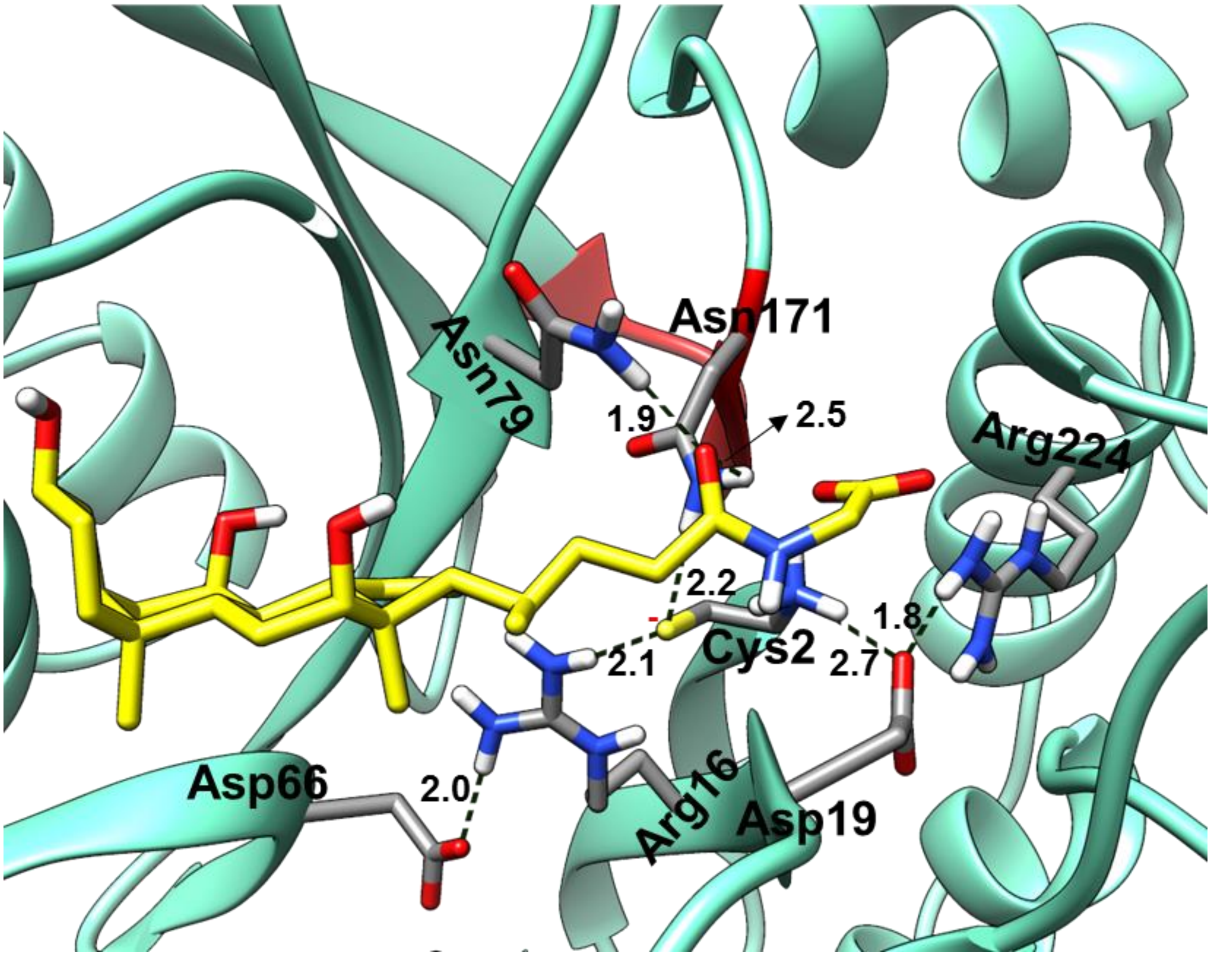

Figure S5. The surface representation of *ls*BSH bound to GCA obtained from the MD simulations. The hydrogen bonds formed by water molecules with the substrate are shown in the black dashed lines. The substrate binding cavity of the *ls*BSH is highlighted with the orange colour. The substrate GCA is shown in yellow colour.

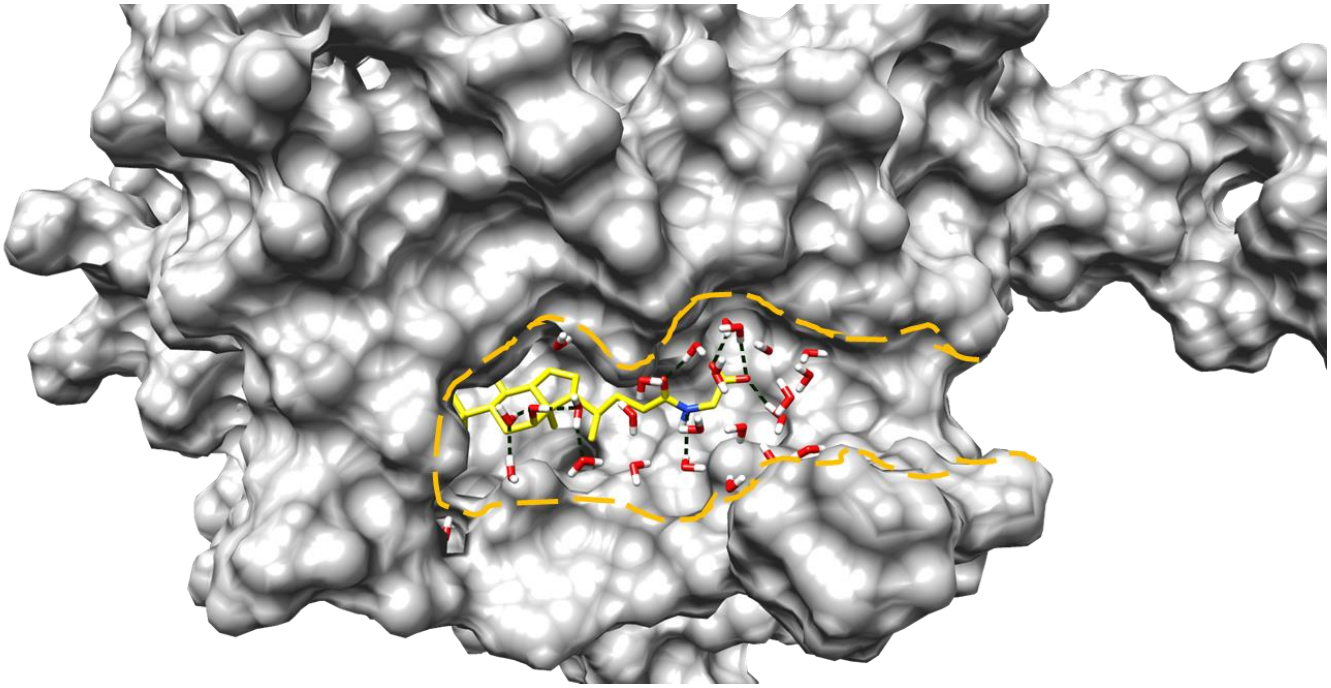

Figure S6. The substrate binding site of *ls*BSH in complex with GCA. The snapshot of the most populated cluster obtained from the 50ns MD simulation. GCA and the substrate stabilizing residues are shown in yellow and grey stick representation, respectively. The hydrophobic interactions mediated by the *ls*BSH residues with the substrate are shown in the orange dashed lines.

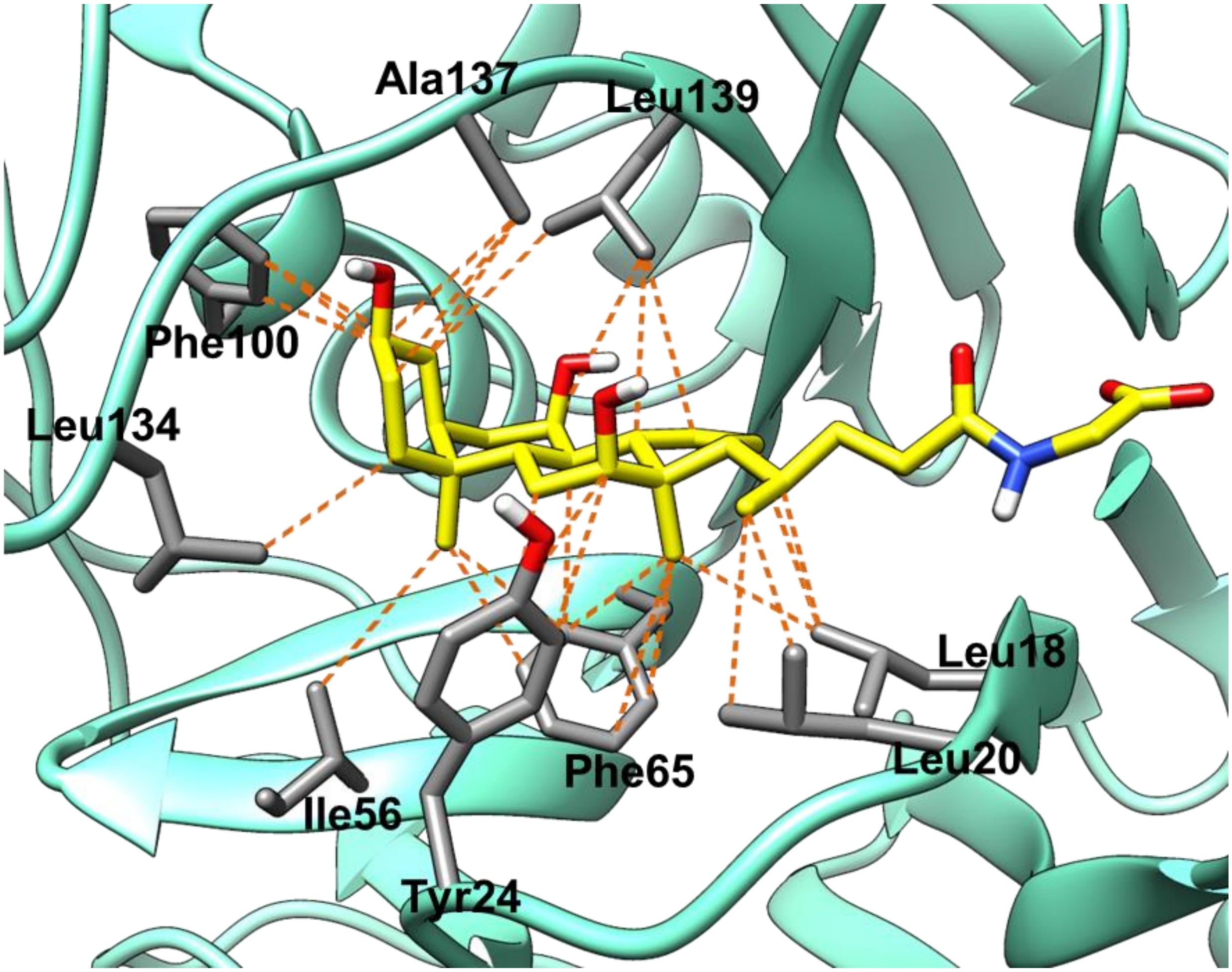

Table S1. Key bacterial plasmids and strains used in this study.

Plasmids or strains	Description	Source or Reference
**Plasmids**		
pBSH	pET21b containing *bsh* gene from *Lactobacillus salivarius* NRRL B-30514, Amp^r	11
pBSH (C2S)	pBSH derivative with C2S mutation	This study
pBSH (Y24F)	pBSH derivative with Y24F mutation	This study
pBSH (F65A)	pBSH derivative with F65A mutation	This study
pBSH (N171A)	pBSH derivative with N171A mutation	This study
pBSH (164-171)	pBSH derivative with the 164 -171 aa motif deleted	This study
pBSH (Q257A)	pBSH derivative with Q257A mutation	This study
pBSH (E270A)	pBSH derivative with E270A mutation	This study
pBSH (Q257A E270A)	pBSH derivative with double mutations (Q257A and E270A)	This study
**Strains**		
E.coli BL21(DE3)	F– ompT gal dcm lon hsdSB(rB- mB-) λ(DE3 [lacI lacUV5-T7 gene 1 ind1 sam7 nin5])	Novagen
E.coli XL1-Blue	EndA1 gyrA96(nal^R) thi-1 recA1 relA1 lac glnV44 F'[::Tn10 $proAB^+$ $lacI^q$ Δ(lacZ)M15] hsdR17(r_K^- m_K^+)	Agilent Technologies
JL885	*E.coli* BL21(DE3) containing the plasmid pBSH	11
JL1189	*E.coli* BL21(DE3) containing pBSH (C2S)	This study
JL1191	*E.coli* BL21(DE3) containing pBSH (Y24F)	This study
JL1193	*E.coli* BL21(DE3) containing pBSH (F65A)	This study
JL1196	*E.coli* BL21(DE3) containing pBSH (N171A)	This study
JL1198	*E.coli* BL21(DE3) containing pBSH (164-171)	This study
JL1201	*E.coli* BL21(DE3) containing pBSH (Q257A)	This study
JL1203	*E.coli* BL21(DE3) containing pBSH (E270A)	This study
JL1205	*E.coli* BL21(DE3) containing pBSH (Q257A E270A)	This study

Figure S7. SDS-PAGE analysis of the purified wild-type (WT) *ls*BSH and its derivative with specific amino acid substitution mutagenesis or deletion.

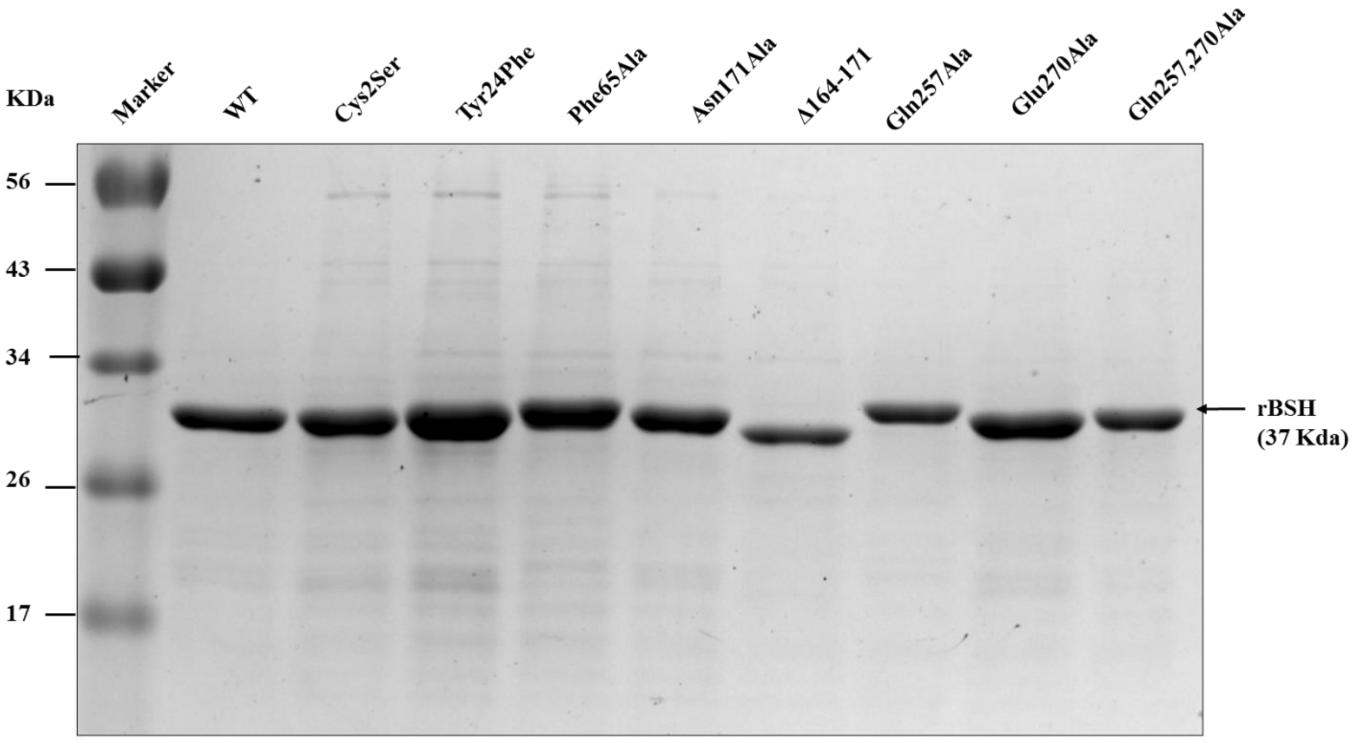

Table S2. The primers used for site-directed amino acid substitution mutagenesis in this study.

Primer Name	Sequence 5'- 3'[a]	AA Mutation
BSHMT1-F	CCATTTAAAGTAATTGCTGT**AGA**CATGGATCCCGACCCATTT	Cys2Ser
BSHMT1-R	AAATGGGTCGGGATCCATG**TCT**ACAGCAATTACTTTAAATGG	
BSHMT2-F	GATTACCTCCTCACC**AAA**TGAAAAATCTAAATCTAAGTTTCTTCCAA	Tyr24Phe
BSHMT2-R	TTGGAAGAAACTTAGA**TTT**AGATTTTTCATTTGGTGAGGAGGTAATC	
BSHMT3-F	TCCATCCTCATTAATAGCATC**TGC**ATACAATGGGTAATCGTTAGCGACAATTCC	Phe65Ala
BSHMT3-R	GGAATTGTCGCTAACGATTACCCATTGTAT**GCA**GATGCTATTAATGAGGATGGA	
BSHMT4-F	CAGATTGTACATCTGATAATTAAATTCTGGATT**TGC**AGTCAATATTCCAATTGG	Asn171Ala
BSHMT4-R	CCAATTGGAATATTGACT**GCA**AATCCAGAATTTAATTATCAGATGTACAATCTG	
BSHMT5-F	GTACATCTGATAATTAAATTCTGGATTATCATAAATATGTACTCCAGATTTAGTTACTTC	Δ164-171
BSHMT5-R	GAAGTAACTAAATCTGGAGTACATATTTATGATAATCCAGAATTTAATTATCAGATGTAC	
BSHMT6_Q257A-F	CCATATACTAGGGACAGTAGAA**GCA**ATAAAGGGCGTTAATAAGACAG	Gln257Ala
BSHMT6_Q257A-R	CTGTCTTATTAACGCCCTTTAT**TGC**TTCTACTGTCCCTAGTATATGG	
BSHMT7_E270A-F	GACAGAATCAGGAAAAGAA**GCA**TACACTGTATATTCGAATTGC	Glu270Ala
BSHMT7_E270A-R	GCAATTCGAATATACAGTGTA**TGC**TTCTTTTCCTGATTCTGTC	

[a] Bolded letters indicate the nucleotides designed for aa substitution mutagenesis

Figure S8. Comparison of the *ls*BSH binding site in the wild type (A) and Tyr24Phe mutation (B). The water-mediated hydrogen bond between Tyr24 and the hydroxyl group of GCA is shown in black dashed lines. The hydrophobic interactions between Phe24 and GCDCA are shown in the orange dashed lines.

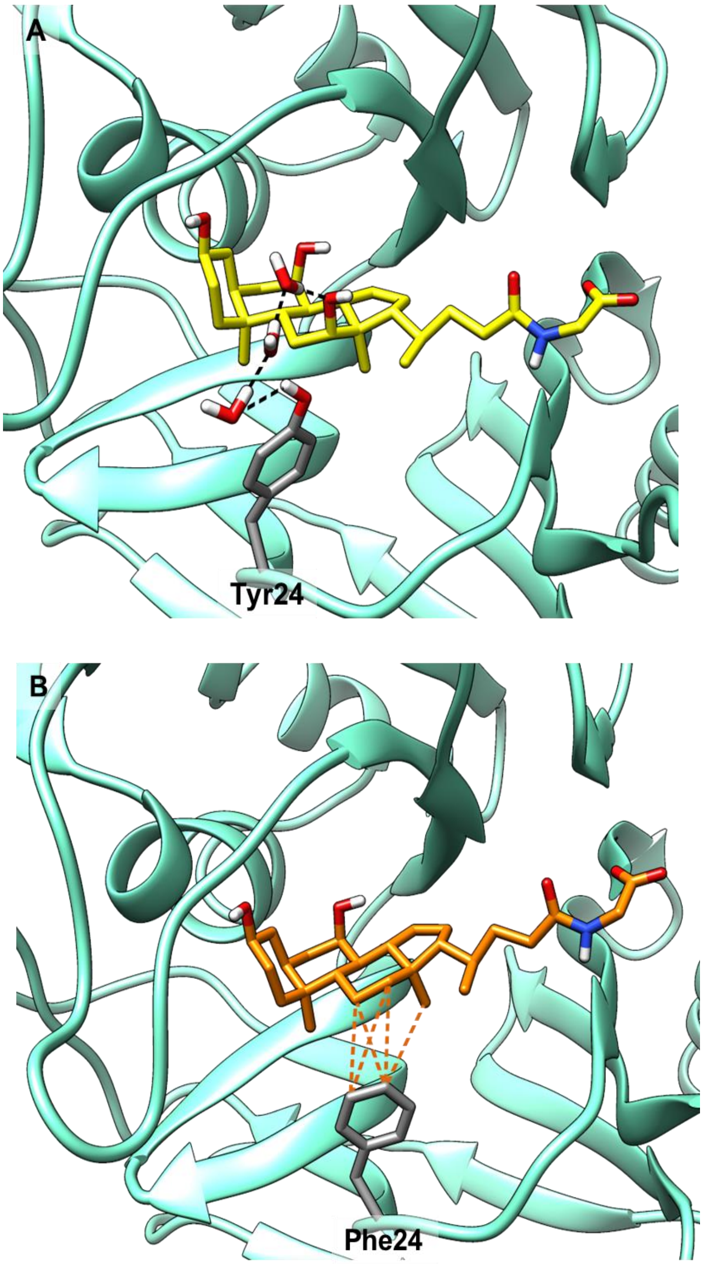

Supplement: Supplementary file 1 — Supplementary Information [file 41598_2019_48850_MOESM1_ESM.pdf]
